# Strain-transcending immune response generated by chimeras of the malaria vaccine candidate merozoite surface protein 2

**DOI:** 10.1038/srep20613

**Published:** 2016-02-11

**Authors:** Bankala Krishnarjuna, Dean Andrew, Christopher A. MacRaild, Rodrigo A. V. Morales, James G. Beeson, Robin F. Anders, Jack S. Richards, Raymond S. Norton

**Affiliations:** 1 Medicinal Chemistry, Monash Institute of Pharmaceutical Sciences, Monash University, Parkville 3052, Australia; 2 Centre for Biomedical Research, Burnet Institute, Melbourne, Victoria 3004, Australia; 3 Department of Microbiology, Monash University, Clayton 3800, Australia; 4 Department of Medicine, University of Melbourne, Parkville 3052, Australia; 5 Department of Biochemistry and Genetics, La Trobe Institute for Molecular Science, La Trobe University, Melbourne, Victoria 3086, Australia

## Abstract

MSP2 is an intrinsically disordered protein that is abundant on the merozoite surface and essential to the parasite *Plasmodium falciparum*. Naturally-acquired antibody responses to MSP2 are biased towards dimorphic sequences within the central variable region of MSP2 and have been linked to naturally-acquired protection from malaria. In a phase IIb study, an MSP2-containing vaccine induced an immune response that reduced parasitemias in a strain-specific manner. A subsequent phase I study of a vaccine that contained both dimorphic forms of MSP2 induced antibodies that exhibited functional activity *in vitro*. We have assessed the contribution of the conserved and variable regions of MSP2 to the generation of a strain-transcending antibody response by generating MSP2 chimeras that included conserved and variable regions of the 3D7 and FC27 alleles. Robust anti-MSP2 antibody responses targeting both conserved and variable regions were generated in mice, although the fine specificity and the balance of responses to these regions differed amongst the constructs tested. We observed significant differences in antibody subclass distribution in the responses to these chimeras. Our results suggest that chimeric MSP2 antigens can elicit a broad immune response suitable for protection against different strains of *P. falciparum*.

Malaria causes over 200 million cases and nearly 650,000 deaths per year worldwide^1^, with the most severe disease caused by *Plasmodium falciparum*. Recent gains in malaria control and progress towards elimination and eventual eradication are under threat because of the rise of drug-resistant parasites^2^ and insecticide-resistant mosquitoes^3^. Eventual global eradication is likely to depend on the development of multi-stage vaccines that prevent or limit blood-stage infection and block ongoing transmission. Previous clinical trials of blood-stage antigens such as merozoite surface protein 2 (MSP2) and apical membrane antigen 1 (AMA1) suggested that vaccine-induced immune responses can reduce the parasite burden, although their efficacy is potentially limited by the antigenic polymorphisms of *Plasmodium* blood-stage antigens^4^,^5^.

MSP2 is a ~23-kDa surface coat protein essential for survival of the asexual blood-stages of *P. falciparum*^6^,^7^,^8^. MSP2 consists of a central variable region (VR) that constitutes 60% of the protein, flanked by conserved N-terminal and C-terminal regions (NTR and CTR, respectively)^9^,^10^. All forms of MSP2 can be grouped into two allelic families, 3D7 and FC27, based on dimorphic sequences within the VR^9^,^10^,^11^,^12^,^13^. These sequences are interspersed by more polymorphic repeat sequences, with the 3D7 allele characterized by highly variable GSA-rich repeats, and FC27-MSP2 harbouring tandem repeats of 32- and 12-residue sequences (Fig. 1A)^9^,^14^,^15^,^16^. MSP2 is highly disordered, lacking any well-defined conformation in solution^17^, and the conformational and antigenic properties of various MSP2 regions are defined entirely by local sequence features^17^,^18^. In particular, all MSP2 epitopes mapped to date consist of short linear sequences^19^.

Anti-MSP2 antibodies are generated following natural infections and correlate with the age-dependent protection observed in adults and children over the age of 5 years^20^,^21^. Anti-MSP2 antibodies are mostly of the IgG1 and IgG3 subtypes and function through antibody-dependent cellular inhibition (ADCI), opsonic phagocytosis and complement-mediated mechanisms^22^,^23^,^24^. The acquisition of anti-MSP2 antibodies alone or in combination with antibodies targeting other *P. falciparum* antigens is an important indicator of a reduced risk of clinical infection^20^,^23^,^25^.

The Combination B vaccine, composed of full-length 3D7 MSP2 (Ag1624), MSP1 (190LCS.T3, N-terminal end, K1 allele) and the C-terminal 70% of RESA (Ag1505H), adjuvanted in Montanide ISA720, reduced parasite densities by 62% when tested in a phase I-IIb trial in Papua New Guinean children^26^. However, the reduction in parasitemia was biased towards parasites containing the 3D7 MSP2 allele^26^,^27^, and consequently a higher incidence of morbidity associated with FC27 MSP2-type parasites was reported. A subsequent phase I trial of a combination of 3D7 and FC27 alleles of full-length MSP2, adjuvanted in Montanide ISA720^24^, exhibited functional activity *in vitro* that included ADCI^24^ and complement-mediated inhibition of parasite growth^22^. These trials suggested that MSP2 vaccines can induce functional responses that may mediate *in vivo* protection, but highlighted the need to evaluate the benefits of including the VR from both alleles, as well as the conserved NTR and CTR.

The functional role of NTR and CTR epitopes is unclear but it would seem logical that targeting these conserved epitopes could mediate strain-transcending immunity. Some of the antibodies to these conserved epitopes do not recognize native MSP2, despite recognizing recombinant MSP2^19^,^28^. Moreover, it has been observed that the NTR can contribute to fibril formation of recombinant MSP2^29^,^30^. It is therefore important to determine which aspects of the NTR and CTR are advantageous in construct design. In order to overcome the allele-specific immune response, it has been proposed that the VR of the two allelic types of MSP2 (3D7 and FC27) should be included in an MSP2 vaccine^24^,^31^. In this study, we sought to determine the effects of immunizing with six truncated and chimeric MSP2 constructs compared to using a combination of both alleles of full-length MSP2^32^. Chimeric antigens also offer the potential advantages of simplified manufacture, simplified quality assurance and reduced costs, which may be particularly important in the context of future multi-component vaccines targeting multiple stages of the *Plasmodium* life cycle^33^.

In this work we have exploited the highly disordered structure of MSP2, in which all epitopes mapped to date consist of short linear sequences^19^, to investigate the roles of the conserved and variable regions of 3D7 and FC27 MSP2 in shaping the antibody repertoire against these proteins. A series of MSP2 constructs was designed to accommodate both allelic forms of the protein as well as permutations in their conserved, polymorphic and repeat regions. These chimeras allowed us to test whether, by manipulating the composition of these chimeras, we could enhance the immune response, target it toward potentially protective epitopes, remove the propensity of MSP2 for aggregation, and modulate the IgG subclass distribution elicited by MSP2.

## Results

### Rationale, design and production of engineered MSP2 constructs

**Figure 1:**
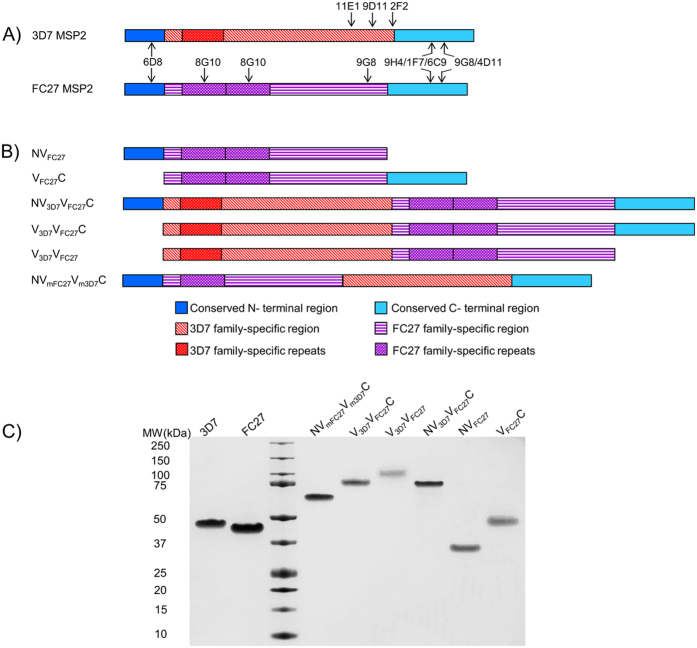
(**A**) Schematic representation of recombinant 3D7 and FC27 MSP2 constructs and the binding regions of previously identified mouse monoclonal antibodies^19^. (**B**) Representation of truncated and chimeric MSP2 constructs used in this work. Truncated constructs were derived from the excision of either conserved N-terminal or C-terminal regions. Chimeric constructs were generated by the fusion of 3D7 and FC27 variable regions in the presence or absence of the conserved N- or C-terminus. (**C**) SDS-PAGE analysis of purified full-length 3D7 MSP2, FC27 MSP2 and other MSP2 constructs: NV_FC27_ - FC27 MSP2 without the CTR (FC27 MSP2_1–171_); V_FC27_C - FC27 MSP2 without NTR (FC27 MSP2_26–221_); NV_3D7_V_FC27_C - NTR, 3D7 MSP2_1–179_ followed by FC27 MSP2_26–171_ (with two 12-residue repeats) and CTR; V_3D7_V_FC27_C- 3D7 MSP2_26–179_ followed by the FC27 MSP2_26–171_ and CTR; V_3D7_V_FC27_- VR of 3D7 MSP2_26–179_ followed by the VR of FC27 MSP2_26–171_; NV_mFC27_V_m3D7_C - NTR, followed by the modified variable regions of FC27 MSP2_64–171_, 3D7 MSP2_58–179_ and CTR. Gel was loaded with 1.25 µg of protein per well and stained with Coomassie Blue.

We designed a set of chimeric antigens (Fig. 1B) consisting of different regions of 3D7 and FC27 MSP2 in order to address two issues: 1) could chimeric MSP2 constructs induce effective antibody responses to both 3D7 and FC27 forms of MSP2, and 2) what impact did the conserved regions have on the immunogenicity and aggregation propensity of MSP2. We name these constructs using a scheme in which the NTR and CTR are denoted as N and C, respectively, and the VR of 3D7 and FC27 are denoted as V_3D7_ and V_FC27_, respectively. Accordingly constructs, NV_FC27_, and V_FC27_C, represent FC27 MSP2 lacking the conserved CTR or NTR, respectively, while NV_3D7_V_FC27_C is a simple chimera containing both VRs flanked by the NTR and CTR, and V_3D7_V_FC27_C and V_3D7_V_FC27_ lack one or both conserved regions, respectively. In NV_mFC27_V_m3D7_C, the order of the VRs is inverted, and the number of repeat regions reduced in order to assess the role of the VR in aggregation propensity as well as the significance of tandem repeat sequences for immunogenicity of other epitopes of MSP2 (Fig. 1B). The complete amino acid sequences of all constructs are given in Table S1.

All constructs were produced in an *E. coli* expression system optimised for high-yield expression of MSP2^18^. The purified proteins migrated as single bands in SDS-PAGE (Fig. 1C) and were pure by analytical HPLC (Fig. S1). All MSP2 constructs used had anomalous relative molecular masses on SDS-PAGE (Fig. 1C), as expected for highly hydrophilic intrinsically disordered proteins^34^. However, LC-MS data confirmed that all MSP2 constructs had the expected molecular mass (Table S2) and purity >99% (Fig. S1). Estimated endotoxin levels were less than 0.1 EU/μg protein (Table S2).

### Protein aggregation is reduced in chimeric constructs

Both allelic forms of full-length recombinant MSP2 are intrinsically disordered and are prone to aggregation and fibril formation in solution^17^,^35^. This aggregation is driven by the conserved NTR of MSP2, but FC27 MSP2 is much more prone to aggregation than 3D7 MSP2, indicating that variable-region sequences also play a role in modulating aggregation propensity^29^,^30^,^35^,^36^. Size-exclusion chromatographic analysis of the six designed MSP2 constructs used in this study detected aggregation only in NV_FC27_ (Fig. S2), which consists of the NTR and the VR of FC27, but lacks the CTR. NV_3D7_V_FC27_C, which contains the conserved NTR followed by 3D7 VR, was not prone to aggregation (Fig. S2), confirming that residues following the NTR modulate aggregation.

### Immunogenicity of the MSP2 constructs

**Figure 2:**
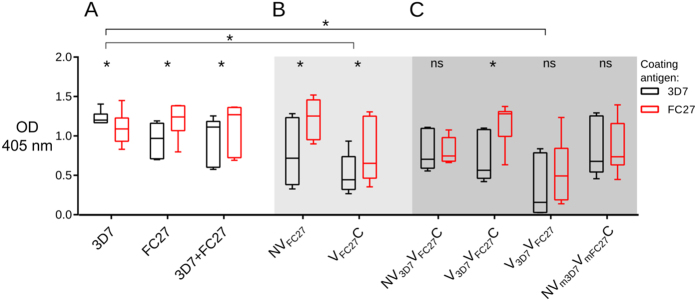
Anti-MSP2 responses of mice immunized with recombinant MSP2 constructs. Total IgG levels tested against 3D7 (black) and FC27 (red) MSP2 measured by ELISA. Responses to 3D7 and FC27 MSP2 were compared with those to the mixture of the two alleles (**A**), to N- and C-terminally truncated FC27 MSP2 (**B**) and to MSP2 chimeras (**C**). The difference in responses to 3D7 and FC27 alleleic variants was compared using the Wilcoxon matched pairs test, while between-group comparisons were made by the Kruskal-Wallis test. * denotes p < 0.05; OD, optical density.

Montanide ISA720 was selected as the adjuvant for the mouse immunogenicity studies because of its use in previous phase I and II studies of MSP2^24^,^37^. Endpoint titres (EPT) were calculated for total IgG responses and compared with a single sera dilution of 1:20,000 to ensure that this sera dilution was within the linear range of the assay. EPT and the single sera dilution at 1:20,000 were highly correlated (Fig. S3), so single sera dilutions were used for the subsequent analyses. IgG levels in sera of mice immunized with the mixture of full-length 3D7 and FC27 MSP2 were not significantly different from those in sera of mice immunized with either 3D7 or FC27 MSP2 alone (Fig. 2A). Unexpectedly, the response generated by the mixture of the two alleles displayed a degree of strain-specificity, with a significantly higher ELISA signal seen on plates coated with FC27 MSP2 than on those coated with 3D7. IgG responses elicited by the C-terminally truncated FC27 construct NV_FC27_ were similar to those elicited by full-length FC27, whereas the N-terminal truncation, V_FC27_C, generated a somewhat weaker response (Fig. 2B).

The majority of the chimeric MSP2 constructs induced total anti-MSP2 IgG levels similar to those induced by immunization with full-length 3D7 or FC27 MSP2 (Fig. 2C). Moreover, in contrast to the response to the mixture of FC27 and 3D7 MSP2, most chimeras generated responses that were not strain-specific. Lower total IgG levels were induced by V_FC27_C and V_3D7_V_FC27_, both of which lacked the conserved NTR. However, V_3D7_V_FC27_C, which also lacks the NTR, induced high IgG levels. V_3D7_V_FC27_ differs from V_3D7_V_FC27_C only in the absence of the conserved CTR, but generates lower IgG responses than V_3D7_V_FC27_C and all other constructs (Fig. 2). These results suggest that both conserved regions are important for the IgG response, albeit in a somewhat context-dependent manner.

### Specificity of the response to a mixture of full-length 3D7 and FC27 MSP2

**Figure 3:**
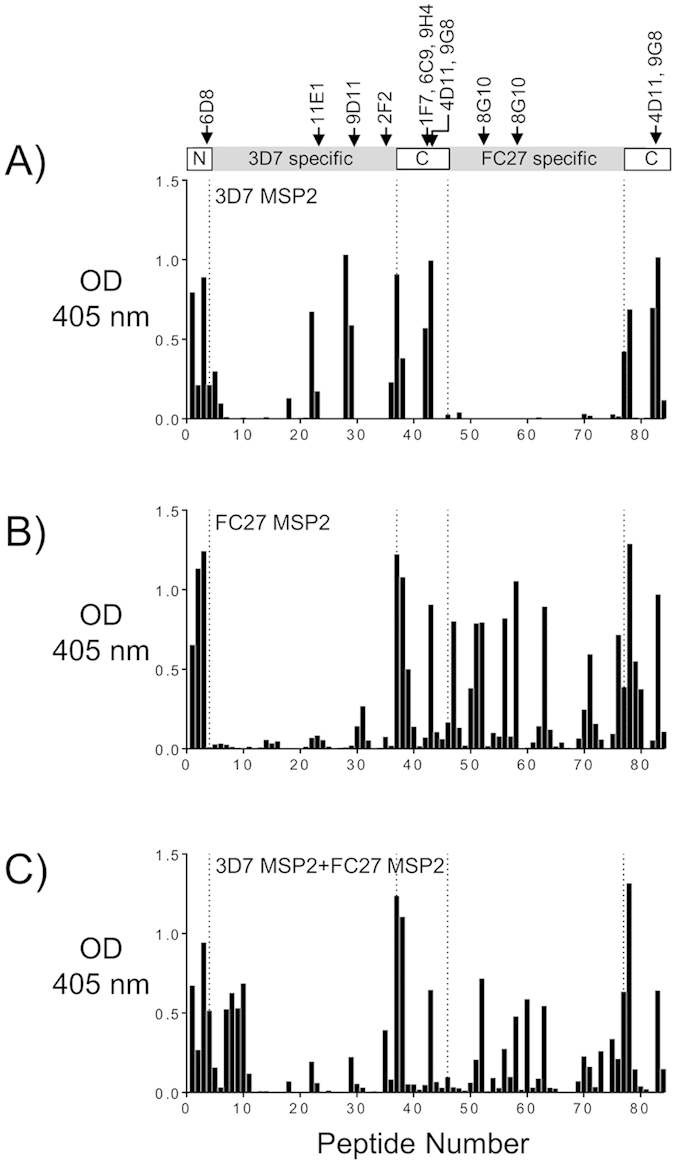
Epitope mapping for 3D7 and FC27 MSP2, and for the mixed immunization, by peptide array using ELISA^19^. Linear epitopes along 3D7 MSP2 and FC27 MSP2 were mapped using pooled sera from mice immunized with recombinant full length 3D7 or FC27 MSP2 and a mixture of 3D7 and FC27 MSP2 (**A**–**C**, respectively). The corresponding epitopes of known mouse mAbs are indicated. Antibody reactivity towards 3D7 and FC27 is uniform towards their conserved and variable regions (**A**,**B**) in contrast to immunizations using a mixture of both alleles which shows broad epitope coverage albeit lower overall reactivity (**C**). In all peptide arrays (including Figs 4 and 5), the small differences in the reactivity to the conserved C-terminal epitopes are due to a small difference between the linear peptide sequences extending from the central 3D7 and FC27 dimorphic regions. OD, optical density.

The epitope specificity of the antibody responses was determined using an overlapping peptide array that included the NTR, VR of both allelic variants, and the CTR. Epitope-specific responses for animals immunized with full-length 3D7 and FC27 (Fig. 3A,B) were in agreement with the pattern of antigenicity described previously in mice, rabbits and in humans^18^,^38^,^39^. Robust responses were observed to conserved epitopes spanning the entire NTR, to multiple epitopes within the VRs of both MSP2 alleles, and to two distinct sequences within the CTR (Fig. 3A–C). The response to the mixture of 3D7 and FC27 MSP2 showed broadly similar specificity to those induced by the two individual immunizations (Fig. 3C). However, although responses to conserved-region epitopes were identical across the three immunization groups, the responses to VR epitopes were somewhat weaker in the 3D7 + FC27 MSP2 immunization group. This was particularly true of the 3D7 MSP2 variable region (except in responses against 3D7-specific repeats, peptides 7–11), consistent with the strain specificity observed in this response (Fig. 3C). Unexpectedly, the response induced by the mixture retained significant strain-specificity for FC27 MSP2.

### The impact of MSP2 conserved regions on the specificity of the antibody response to variable region epitopes

**Figure 4:**
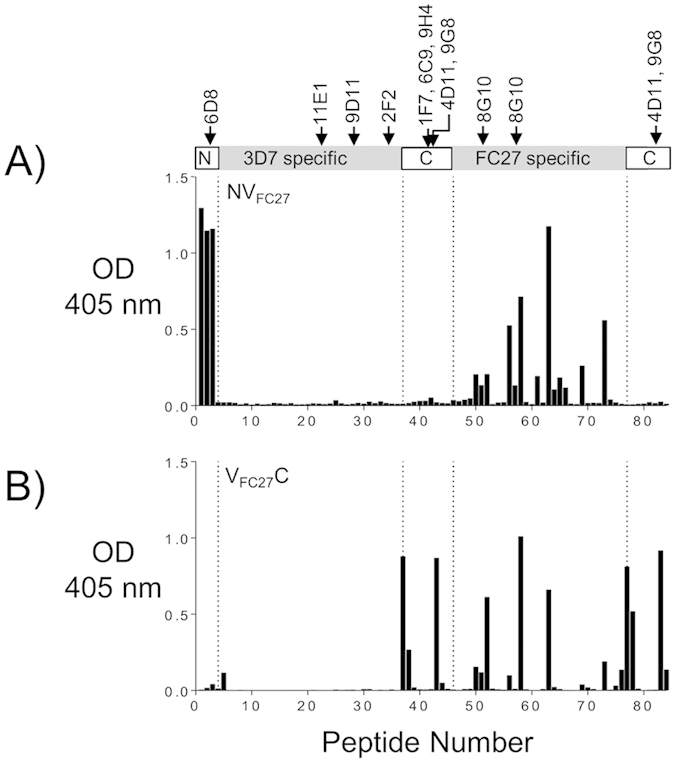
Epitope mapping for NV_FC27_ and V_FC27_C constructs by peptide array using ELISA^19^. Linear epitopes along 3D7 MSP2 and FC27 MSP2 were mapped by peptide array for sera from mice immunized with NV_FC27_ and V_FC27_C (**A,B** respectively) constructs. The respective regions of known mouse mAbs are indicated in the schematic. Epitope mapping assays were conducted using pooled mice sera from each of the immunization groups. The overall reactivity to the VRs is reduced when compared to the reactivity to the full-length FC27 MSP2 (Fig. 3B). OD, optical density.

Removal of the conserved regions of MSP2 resulted in reduced immunogenicity of the FC27 MSP2 constructs, as measured by total IgG levels, with this reduction being more marked for the construct lacking the NTR (Fig. 2B). To examine this effect in more detail, epitope mapping of responses from mice immunized with each of the chimeras was also undertaken using the peptide array. The antibody responses evoked by FC27 MSP2 constructs lacking either the N- or C-terminal region (i.e. V_FC27_C and NV_FC27_) were broadly similar (Fig. 4), but these responses were weak compared to the strong response to numerous VR epitopes induced by full-length FC27 MSP2 (compare peptides 47–76 in Figs 3B and 4). This result suggests that both the N- and C- terminal conserved regions are necessary to induce robust antibody responses to the VR, at least in the context of FC27 MSP2.

### Chimeric MSP2 antigens elicit antibody responses to both conserved and variable region epitopes

**Figure 5:**
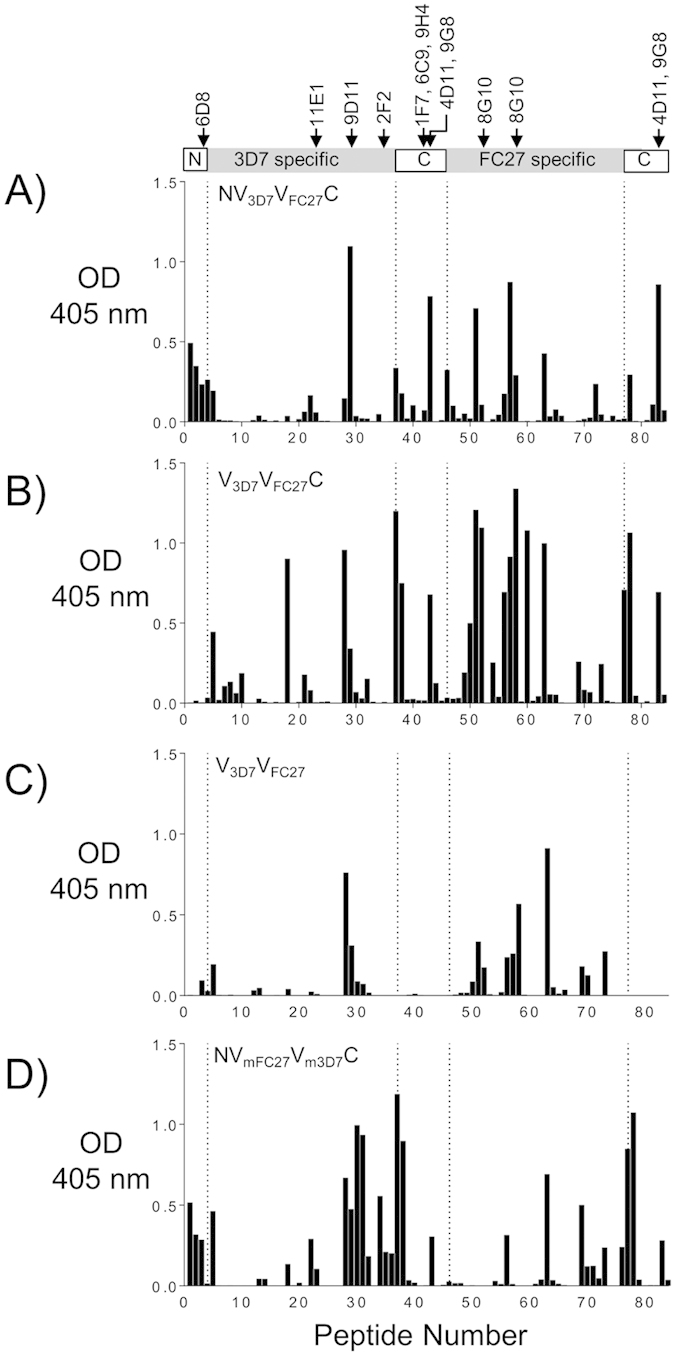
Epitope mapping for MSP2 chimeras by peptide array using ELISA^19^. Linear epitopes along 3D7 MSP2 and FC27 MSP2 were mapped for sera from mice immunized with MSP2 chimeric constructs. The corresponding epitopes of known mouse mAbs are indicated. The overall reactivity to the 3D7 and FC27 epitopes is comparable for the NV_3D7_V_FC27_C (**A**) and improved for the V_3D7_V_FC27_C construct (**B**). The reactivity is reduced for V_3D7_V_FC27_ (C), which is lacking conserved NTR and CTR, and improved and reduced, respectively, for the 3D7 and FC27 VR regions when the GSA repeats and 32-residue repeats were removed in the chimera NV_mFC27_V_m3D7_C (**D**). OD, optical density.

The chimeric 3D7-FC27 MSP2, NV_3D7_V_FC27_C, elicited antibody responses against epitopes in both the NTR and CTR as well as epitopes in the VR of both alleles (Fig. 5A). In contrast to the antibodies elicited by immunization with the mixture of 3D7 and FC27 MSP2, which reacted more strongly with conserved epitopes (Fig. 3C), the strongest antibody responses to NV_3D7_V_FC27_C were to variable region epitopes. Thus, the response induced by the chimeric antigen NV_3D7_V_FC27_C appeared to offer an improved balance between conserved and variable region epitopes.

Removal of the N-terminal conserved region was anticipated to decrease the aggregation and fibril-formation tendencies of MSP2 (Fig. S2), although we found that it also affected the response to the FC27-specific epitopes in V_FC27_C (Fig. 4B). Accordingly, we examined the effects of removal of that region from the chimeric antigen (Fig. 5B). In striking contrast to the results with the N-terminally deleted FC27 MSP2 (V_FC27_C), the antibody response to the chimeric antigen lacking the N-terminus (V_3D7_V_FC27_C) showed improved reactivity with epitopes in the VR of both 3D7 and FC27 MSP2, as well as in the CTR (compare Figs 4B and 5B). These results indicate that removal of the conserved NTR can not only minimize the tendency for protein aggregation but, in this construct, also enhance antibody responses to the VR and CTR.

To further examine the role of conserved regions in the development of a robust antibody response, we immunized animals with V_3D7_V_FC27_, which consists of only the VR regions of both 3D7 and FC27. The resulting sera showed significantly reduced reactivity to the epitopes in the VR (Figs 2C and 5C), confirming the importance of the CTR for a robust antibody response against MSP2. Finally, we examined the response to NV_mFC27_V_m3D7_C, in which the two VRs were presented in the opposite order, relative to the previous chimeras, and in which the tandem repeat regions were truncated (Fig. 1, Table S1). The observed response was similar in magnitude and fine specificity to that seen for NV_3D7_V_FC27_C (Figs 2C and 5D). However, truncation of one 32-residue repeat sequence in FC27 VR supressed the immunogenicity of the remaining 32-residue repeat sequence (peptides 50–62, Fig. 5D), while removal of the complete GGSA repeat sequence of 3D7 VR improved the immunogenicity of the remaining 3D7 VR epitopes. This suggests that the number of 32-residue repeats in FC27 MSP2 predominantly affects reactivity to epitopes encoded by the 32-mer, whereas the GGSA repeat sequence in 3D7 MSP2 may compromise the immunogenicity of other 3D7 VR epitopes.

### IgG subclass responses to different MSP2 constructs

**Figure 6:**
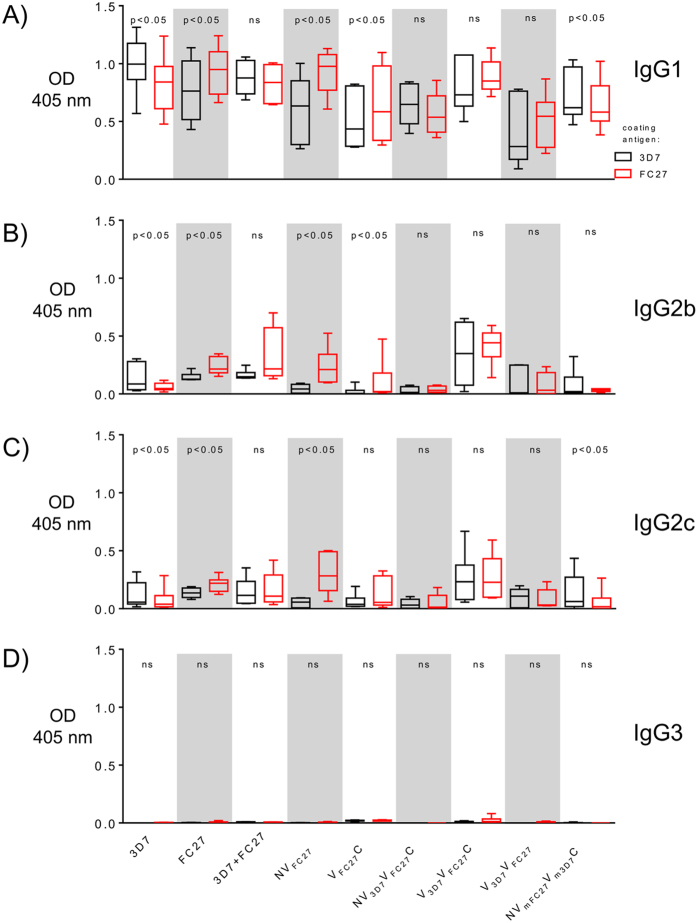
Anti-MSP2 IgG subtype (**A–D** corresponding IgG1, IgG2b, IgG2c and IgG3, respectively) responses of mice sera following immunization with recombinant 3D7, FC27 and other MSP2 constructs. Total IgG subclass levels measured by ELISA against 3D7 MSP2 (black), FC27 MSP2 (red) coated antigens using sera from mice immunized with full-length recombinant 3D7, FC27 and other MSP2 constructs. Data shown here are for sera diluted at 1:20,000. Five mice for NV_3D7_V_FC27_C construct and six mice for all other constructs were included in each of the immunization groups, with the mean and standard deviation indicated. IgG1 and IgG2b response was biased towards FC27 MSP2 for FC27 MSP2, NV_FC27_ and NV_3D7_V_FC27_C constructs, while the IgG1 response was biased towards 3D7 MSP2 and for the 3D7 MSP2 and NV_mFC27_V_m3D7_C. IgG1 and IgG2c response was biased towards the 3D7 MSP2 for the NV_mFC27_V_m3D7_C construct. There was no difference in the IgG3 subclass observed. On the other hand, more balanced IgG subclasses were observed for the mixture of 3D7 and FC27 MSP2, NV_3D7_V_FC27_C, V_3D7_V_FC27_C and V_3D7_V_FC27_ constructs despite the presence or absence of the conserved NTR and CTR. The difference in responses to 3D7 and FC27 allelic variants was compared using the Wilcoxon Matched Pairs Test. OD, optical density.

Human anti-MSP2 antibodies may mediate protection by Fc receptor-mediated processes including ADCI^24^ and opsonic phagocytosis^23^, and by complement-mediated growth inhibition^22^. Therefore it is likely that a human MSP2-based malaria vaccine will need to induce and sustain high titres of IgG1 and/ or IgG3 antibodies, which mediate these effector mechanisms, as is seen with naturally-acquired anti-MSP2 responses^20^,^40^,^41^. IgG1 was the dominant subclass response to all MSP2 constructs, including the four chimeras studied here, in mice (Fig. 6A), but, in contrast to human IgG1, murine IgG1 effector functions are not mediated by interactions with Fc receptors or complement. Lower levels of IgG2b and IgG2c antibodies were induced by the MSP2 constructs although the highest IgG2b and IgG2c responses were observed in mice immunized with V_3D7_V_FC27_C (Fig. 6B,C). These murine subclasses, which share characteristics with human IgG3 and IgG1, respectively, are cytophilic and complement fixing^42^. IgG3 responses were negligible (Fig. 6D).

### Chimeric MSP2 antigens elicit antibodies capable of strain-independent recognition of parasite extracts

**Figure 7:**
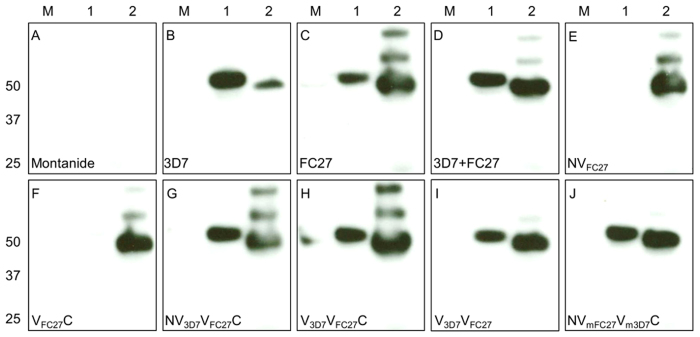
Western blot analysis of the Reactivity of mice sera against *P. falciparum* lysates following immunization with MSP2 constructs. Imunization with different MSP2 constructs promoted a strong antibody response that recognized the native MSP2 in 3D7 and FC27 (D10 clone) parasite extracts. Removal of either the conserved N-terminal or C-terminal regions of FC27 MSP2 abolished the cross-reactivity to the 3D7 parasite (NV_FC27_ and V_FC27_C). The labels at the left bottom side of the gel indicate the corresponding constructs used in mice immunization experiments. M = protein marker and 1 and 2 indicated the 3D7 and FC27 (D10 clone) parasite extracts, respectively. Panels (**C**–**H**) show that antibodies cross-react with aggregated forms of FC27, albeit more weakly than with the monomeric species. (Panel **I**) shows that this cross-reactivity is even weaker in the absence of the conserved N- and C-terminal conserved regions. It is also suppressed in the NV_mFC27_V_m3D7_C chimera (panel **J**).

An important requirement of a protective immune response against MSP2 is the ability to recognize the protein on the parasite surface. We have characterized several epitopes on recombinant MSP2 that appear to be significantly less accessible on the parasite surface^19^, and have shown that the masking of these epitopes is recapitulated in parasite-derived material examined by western blot^19^. Accordingly, we have employed this approach to test the antisera raised against each of our MSP2 constructs, using parasite strains expressing 3D7 and FC27 MSP2 (Fig. 7). The antisera raised against individual MSP2 alleles showed cross-reactivity in western blot, indicating that, despite the extensive masking of conserved region epitopes, some antibodies to the conserved regions are able to recognize MSP2 on the parasite surface. In contrast, no such cross-reactivity is observed for the truncated versions of FC27 MSP2 lacking either the conserved N- or C-terminal regions, despite the presence of antibodies reactive to the remaining conserved region in each case, as judged from the peptide array data (Fig. 4) and the cross-reactivity of total IgG and IgG subtypes (Figs S4 and S5). This suggests that generating antibodies to the conserved epitopes of MSP2 on the parasite requires the presence of both conserved regions, at least in the context of FC27 MSP2 (Fig. 7C, E and F). Antibodies elicited by the mixture of 3D7 and FC27 MSP2 allelic forms, as well as by all chimeric constructs, robustly recognize parasites of both strains, confirming that either approach represents an effective strategy for the generation of strain-transcending antibody responses to native MSP2.

## Discussion

The phase IIb Combination B vaccine trial strongly suggested that immune responses against MSP2 are protective^26^,^27^,^43^. The strain-specific protection observed in that study indicated that epitopes within the central variable regions are likely to mediate such responses and that vaccine formulations will need to include multiple MPS2 alleles to afford protection against different parasite strains^26^,^44^. Fortunately, epitopes in the variable region are known to be naturally immunogenic^21^ and much of the variability is dimorphic^13^, suggesting that a bivalent vaccine may be sufficient to cover the natural antigenic diversity of MSP2. In a subsequent phase I study, a mixture of two representative allelic forms of MSP2^24^ induced antibody responses to the central variable region as well as to conserved C-terminal epitopes. Antibodies from this study were shown to inhibit *in vitro* parasite growth when acting in conjunction with complement or monocytes^22^,^24^, suggesting that responses to C-terminal epitopes may also be functionally important. Our study sought to determine if vaccine delivery would be more effective using two recombinant antigens or a chimeric vaccine.

Studies of chimeric antigens based on other vaccine candidates have demonstrated that antigenic diversity can effectively be addressed using this approach^45^,^46^. Both strategies of immunization (chimeric antigens and a mixture of antigens) have yielded evidence for distinct antibody specificity and, in some cases, distinct functional responses, compared with those elicited by monovalent antigens^45^,^46^,^47^,^48^. Nonetheless, the relative merits of these two strategies are not well understood. The series of immunizations described in our study has been designed to assess these issues in the context of MSP2, and to evaluate the significance of the conserved NTR and CTR for the immunogenicity and aggregation propensity of MSP2.

One key advantage proposed for chimeric antigens over multivalent vaccines arises from the simpler production, formulation, quality assurance process and reduced costs likely to be associated with the development of a single chimeric construct. This is expected to be particularly significant in the context of a future multi-component malaria vaccine. On the other hand, the yields of some chimeric constructs are dramatically lower than their constituent antigens^45^, presumably due to challenges associated with the correct folding of structured antigens in the context of larger chimeras. In this respect, the highly disordered nature of MSP2, which obviates the need for folding, represents a key advantage. Indeed, the yields of all constructs examined here are comparable to those of recombinantly expressed 3D7 and FC27 MSP2. Moreover, the specificity of the antibody response across all of these constructs is consistent with our previous observations^18^. We observed that antibody responses to all constructs were focused on a relatively small number of epitopes corresponding to regions of MSP2 that we and others have shown to be particularly immunogenic^18^,^32^,^38^,^49^. This result suggests that the conformational and antigenic properties of MSP2 are for the most part preserved in the context of these chimeras. However, we did observe some unexpected and context-dependent effects with the inclusion of the NTR and CTR on the overall immunogenicity of these chimeras and the fine specificity of the antibody response they elicit. The basis of these effects is unclear, but they may be mediated by T-cell epitopes present within the conserved regions of MSP2^50^,^51^.

For the structured antigen AMA1, immunization with mixtures of diverse alleles elicited a bias towards conserved region epitopes in rabbits^48^,^52^. That was rationalised in terms of the relative dilution of polymorphic epitopes, and its impact on B-cell affinity maturation against AMA1^52^,^53^. The desirability of this effect will ultimately reflect a trade-off between the extent of antigenic diversity on the one hand, and the relative functional capacities of antibodies to conserved and variable epitopes on the other. In the case of MSP2, most variable region epitopes are essentially dimorphic and so are effectively covered by the two major allelic forms. Epitopes in the MSP2 variable region appear to be important targets for protective immunity, while many conserved region epitopes are masked on the parasite surface and therefore unlikely to play a protective role^19^,^28^. However, the importance of conserved MSP2 epitopes to functional antibody responses is yet to be established.

MSP2 has a propensity to form fibrillar aggregates^29^,^35^. While aggregated forms of some antigens are more immunogenic than their monomeric forms^54^,^55^,^56^, aggregated antigens may also generate low-affinity or autoreactive antibodies^57^,^58^. Our MSP2 constructs showed reduced aggregation propensity compared to the native protein, presumably a consequence of the low fibril propensity imposed by the arrangement of the MSP2 sequences in the constructs used in this work. For example, aggregation is abolished in NV_3D7_V_FC27_C and NV_mFC27_V_m3D7_C where the NTR is followed by 3D7 VR and a stretch of residues from the 3D7 VR, respectively. This supports the conclusion that residues immediately C-terminal to the conserved N-terminal region in native FC27 MSP2 contribute to its aggregation. Furthermore, it demonstrates that aggregation can be ameliorated in chimeric MSP2 antigens by appropriate construct design, and represents another advantage of the chimeric antigens over a mixture of individual antigens.

Variation in the number of tandem repeat sequences could affect their immunogenicity^14^. This is further confirmed in this work, where the removal of one 32-residue repeat diminished the immune response to the other 32-residue repeat in NV_mFC27_V_m3D7_C. Surprisingly, in the same construct the removal of 3D7 allele-specific GSA-rich sequences improved the response to the epitopes in 3D7 MSP2 dimorphic region. This suggests that the GSA repeats may modulate the immunogenicity of the epitopes in the 3D7 MSP2 dimorphic region and may explain why 3D7 MSP2 is less immunogenic than FC27 MSP2 in the mixture of the two antigens.

Anti-MSP2 antibodies associated with protection in natural infections are mainly IgG1 and IgG3 subtypes^11^,^20^,^40^,^41^,^43^, with IgG1 predominant in children and IgG3 predominant in older malaria-exposed individuals^41^. These cytophilic subclass responses are likely to be important in eliciting complement- and monocyte-mediated effector responses. The IgG subtypes elicited by the MSP2 constructs tested in this work are biased mainly towards murine IgG1 and IgG2b subclasses. Murine IgG2b shares many features with human IgG3^42^. In our experiments, the chimeric construct V_3D7_V_FC27_C was able to induce strong antibody responses reactive to the conserved C-terminal and VR region epitopes of both 3D7 and FC27 MSP2 alleles. This construct also provided a more balanced IgG subtype response, shifting the dominance of IgG1 towards IgG2b without significantly affecting the total IgG titre. A better understanding of the mechanisms responsible for protection by antibodies targeting MSP2^22^,^23^ and of the IgG subclasses responsible for those effector responses will enable the design of optimal MSP2-based constructs for inclusion in a future malaria vaccine.

These results demonstrate that our chimeric approach enables both the fine epitope specificity and the distribution of IgG subclasses to be optimised. The V_3D7_V_FC27_C chimera was able to induce IgG levels that were the equivalent of that obtained from immunizing with co-administered 3D7 + FC27, but was able to induce stronger IgG2b and IgG2c responses. This chimera induced strong responses against the 3D7- and FC27-specific epitopes, as well as strong response to the C-terminal conserved epitopes. The V_3D7_V_FC27_C chimera demonstrates that such approaches can achieve responses that are equivalent to or better than those of admixed recombinant antigens, while providing potential advantages in manufacture and cost.

## Methods

### Expression of MSP2 chimeras and truncated FC27 MSP2 proteins

Full-length 3D7 MSP2 (JN248383) and FC27 MSP2 (JN248384) alleles (Fig. 1A) expressed in *E. coli* to GMP standards were available from a recent phase I clinical trial^24^. Codon-optimized gene sequences for six MSP2 constructs (truncated FC27 MSP2 and chimeras) were obtained from GenScript (Fig. 1B), as follows: NV_FC27_ - FC27 MSP2 without the CTR (FC27 MSP2_1–171_); V_FC27_C - FC27 MSP2 without NTR (FC27 MSP2_26–221_); NV_3D7_V_FC27_C - NTR, 3D7 MSP2_1–179_ followed by FC27 MSP2_26–171_ (with two 12-residue repeats) and CTR; V_3D7_V_FC27_C- 3D7 MSP2_26–179_ followed by the FC27 MSP2_26–171_ and CTR; V_3D7_V_FC27_- VR of 3D7 MSP2_26–179_ followed by the VR of FC27 MSP2_26–171_; NV_mFC27_V_m3D7_C - NTR, followed by the modified variable regions of FC27 MSP2_64–171_, 3D7 MSP2_58–179_ and CTR. The amino acid sequences of all MSP2 constructs are shown in Table S1.

Synthetic genes were sub-cloned into a pET32a vector at KpnI and NcoI restriction sites for expression as thioredoxin-fusion proteins. The sequence of each fusion protein was confirmed by DNA sequencing. MSP2 plasmids were transformed into *E. coli* BL21 (DE3) cells and protein expression was carried out at 37 °C for 3 h in the presence of 1 mM isopropyl β–D-1-thiogalactoside. The cells were then harvested by centrifugation at 6000 rpm for 15 min and the cell pellets were stored at −80 °C until protein purification.

### Protein purification

Bacterial cell pellets were resuspended in 20 mM Tris (pH 8.0) buffer containing 100 mM NaCl and 20 mM imidazole in the presence of protease inhibitors (Roche Life Sciences) and lysed by boiling for 10 min^59^. The fusion proteins were purified by affinity chromatography as described earlier^17^,^18^. Following TEV cleavage of the fusion proteins at 34 °C overnight, the His-tagged thioredoxin was removed by affinity chromatography. The flow-through containing MSP2 was further purified by anion-exchange chromatography and reverse-phase high-pressure liquid chromatography (RP-HPLC). HPLC purification was carried out using a C18 column (250 mm × 10 mm, ZORBAX) equilibrated with solvent A (0.1% TFA in water) and the proteins were loaded at a flow rate of 2 mL/min. The bound proteins were eluted using a linear gradient of acetonitrile (solvent B: 80% acetonitrile/0.1% TFA) over 45 min. The HPLC fractions containing protein were analysed by SDS-PAGE and LC-MS. Final yields of all purified proteins ranged from 2–10 mg/L of bacterial culture. The purified proteins were lyophilized and stored at −80 °C until further use.

### Aggregation propensity of MSP2 constructs

The aggregation propensities of all MSP2 constructs were characterized by size-exclusion chromatography prior to immunization experiments. Each construct was incubated at l mg/mL in 25 mM potassium phosphate buffer (pH 6.5) for four weeks at 4 °C prior to testing for aggregation. Samples were also tested after heating at 90 °C for 10 min just before loading onto the size-exclusion column. For size-exclusion chromatography a Superdex-75 column (GE) equilibrated with 25 mM of potassium phosphate buffer was used with a flow rate of 0.5 mL/min.

### Protein preparation and mice immunization experiments

Full-length 3D7 and FC27 MSP2 and the six designed MSP2 constructs were reconstituted as stock solutions of 1 mg/mL in 10 mM phosphate buffered saline (PBS) and tested for the presence of endotoxin using a Limulus Amebocyte Lysate (LAL) assay kit (GenScript). The kit has a minimum detection limit of 0.005 EU/mL. In this work, the maximum acceptable endotoxin value was set to 0.1 EU/μg protein^60^. Protein stock solutions were stored at −80 °C until further use.

Prior to immunizations, MSP2 stock solutions were diluted to 0.35 mg/mL in PBS, heated at 100 °C for 5 min, cooled, then filtered through a 0.02 μm filter to remove MSP2 aggregates. All forms of MSP2 were formulated with Montanide ISA720 at 3:7 ratio (antigen:adjuvant) to a final concentration of 0.1 mg/mL. Female C57BL/6 mice (n = 5 per group for NV_3D7_V_FC27_C and n = 6 per group for other constructs) were inoculated subcutaneously with 100 μL containing 10 μg of formulated antigen at weeks 0, 4 and 8, then euthanized at week 10. The 3D7 MSP2 + FC27 MSP2 group received 10 μg of each antigen (i.e. 20 μg total protein). Sera were collected and stored at −20 °C. All procedures were approved by AMREP AEC (Approval No. E/1417/2013/F) and all methods were carried out in accordance with the approved guidelines.

### Antibody characterization with a peptide array

The specificity profile of antibodies induced by immunization with each MSP2 construct was characterized using a peptide array composed of 84 biotinylated 13-mer peptides, with an 8-residue overlap, covering the entire 3D7 and FC27 MSP2 sequences, as described earlier^18^,^19^. Nunc MaxiSorp flat-bottom 96-well plates were coated with 1 μg/mL streptavidin in PBS and incubated overnight at 4 °C. Plates were washed with PBS containing 0.05% Tween-20, blocked with 0.5% BSA at 37 °C for 2 h and coated with 1:500 biotinylated peptides. Sera samples were pooled for each immunization group, diluted 1:1000 in blocking solution and added to the peptide array. Antibody detection was performed by the addition of goat anti-mouse IgG-HRP and developed using ABTS (2. 2′-azino-bis[3-ethyl benzenethiazoline-6-sulfonic acid]). The colour reaction was quenched by the addition of 1% SDS solution and the optical density was determined at 405 nm. Sera from mice inoculated with adjuvant alone were used as the background control.

### Quantification of total IgG and IgG subclasses

Anti-MSP2 IgG was quantified by ELISA using Nunc MaxiSorp flat-bottom 96-well plates coated with full-length recombinant MSP2 (3D7, FC27) and the other MSP2 constructs at 1.25 μg/mL overnight at 4 °C. Plates were washed with PBS containing 0.05% Tween-20 and blocked with 0.1% casein at 37 °C for 2 h. Sera collected from individual mice were serially diluted to calculate total IgG endpoint titres (EPT) for the two full-length MSP2 antigens and the six designed MSP2 constructs. These EPT were compared with a single sera dilution of 1:20,000 to ensure that this sera dilution was within the linear range of the assay. EPT and the single sera dilution at 1:20,000 were highly correlated (Fig. S3), so single sera dilutions tested in duplicate were used for the subsequent analyses. Total IgG and IgG subclasses were quantified using goat anti-mouse IgG-HRP, IgG1-HRP, IgG2b-HRP, IgG2c-HRP and IgG3-HRP diluted 1:2,000 in blocking buffer. HRP activity was visualised with ABTS read at 405 nm. Sera from mice inoculated with adjuvant alone were used as the background control. A titration of pooled sera from MSP2 immunized mice was added to each plate to allow normalisation of values across different plates.

### Antibody reactivity with parasite lysate

*P. falciparum* 3D7 and FC27 (D10 clone) strains were cultured in human erythrocytes at a hematocrit of 3% in RPMI media supplemented with 10% AlbuMAX II. Infected erythrocytes were harvested at the schizont stage of parasite development at 10% parasitaemia, then lysed with 0.1% saponin. Parasite lysates were fractionated by SDS-PAGE, then transferred to nitrocellulose membranes. Blots were blocked with 5% skim milk, incubated with a 1:500 dilution in 5% skim milk of pooled sera from each group of immunized mice, washed with PBS containing 0.05% Tween-20 (PBS-T), incubated with 1:5,000 goat anti-mouse IgG HRP, washed with PBS-T and developed with Amersham ECL Western Blot detection kit (GE Healthcare).

### Statistical analysis

Total IgG and IgG subclasses (IgG1, IgG2b, IgG2c, IgG3) from each group were compared statistically between recombinant 3D7 and FC27 MSP2 coated ELISA plates using Wilcoxon matched pairs signed rank test. Tests were performed one-sided when there was an expectation of specificity, and two-sided otherwise. Additionally, the Kruskal-Wallis test with Dunn’s post test was performed for each immunization group against recombinant 3D7, FC27 MSP2 and designed MSP2 constructs across total IgG and IgG subclasses.

## Additional Information

**How to cite this article**: Krishnarjuna, B. *et al.* Strain-transcending immune response generated by chimeras of the malaria vaccine candidate merozoite surface protein 2. *Sci. Rep.* **6**, 20613; doi: 10.1038/srep20613 (2016).

## Supplementary Material

Supplementary Information

## References

[b1] WHO. World malaria report. 1–242 (2014).

[b2] Tun K. M. *et al.* Spread of artemisinin-resistant *Plasmodium falciparum* in Myanmar: a cross-sectional survey of the K13 molecular marker. Lancet Infect. Dis. 15, 415–421 (2015). 25704894 10.1016/S1473-3099(15)70032-0 PMC4374103

[b3] Nkya T. *et al.* Insecticide resistance mechanisms associated with different environments in the malaria vector *Anopheles gambiae*: a case study in Tanzania. Malar. J. 13, 28 (2014). 24460952 10.1186/1475-2875-13-28 PMC3913622

[b4] Barry A. E. & Arnott A. Strategies for designing and monitoring malaria vaccines targeting diverse antigens. Front. Immunol. 5, 1–16 (2014). 25120545 10.3389/fimmu.2014.00359 PMC4112938

[b5] Ferreira M. U., da Silva Nunes M. & Wunderlich G. Antigenic diversity and immune evasion by malaria parasites. Clin. Diagn. Lab. Immunol. 11, 987–995 (2004). 15539495 10.1128/CDLI.11.6.987-995.2004 PMC524792

[b6] Smythe J. A. *et al.* Identification of two integral membrane proteins of *Plasmodium falciparum*. Proc. Natl. Acad. Sci. USA 85, 5195–5199 (1988). 3293051 10.1073/pnas.85.14.5195 PMC281715

[b7] Sanders P. R. *et al.* A set of glycosylphosphatidyl inositol-anchored membrane proteins of *Plasmodium falciparum* is refractory to genetic deletion. Infect. Immun. 74, 4330–4338 (2006). 16790807 10.1128/IAI.00054-06 PMC1489731

[b8] Gilson P. R. *et al.* Identification and stoichiometry of glycosylphosphatidylinositol-anchored membrane proteins of the human malaria parasite *Plasmodium falciparum*. Mol. Cell. Proteomics 5, 1286–1299 (2006). 16603573 10.1074/mcp.M600035-MCP200

[b9] Fenton B. *et al.* Structural and antigenic polymorphism of the 35- to 48-kilodalton merozoite surface antigen (MSA-2) of the malaria parasite *Plasmodium falciparum*. Mol. Cell. Biol. 11, 963–971 (1991). 1990294 10.1128/mcb.11.2.963-971.1991 PMC359759

[b10] Smythe J. A. *et al.* Structural diversity in the 45-kilodalton merozoite surface antigen of *Plasmodium falciparum*. Mol. Biochem. Parasitol. 39, 227–234 (1990). 2181307 10.1016/0166-6851(90)90061-p

[b11] Taylor R. R., Smith D. B., Robinson V. J., McBride J. S. & Riley E. M. Human antibody response to *Plasmodium falciparum* merozoite surface protein 2 is serogroup specific and predominantly of the immunoglobulin G3 subclass. Infect. Immun. 63, 4382–4388 (1995). 7591074 10.1128/iai.63.11.4382-4388.1995 PMC173623

[b12] Gerold P., Schofield L., Blackman M. J., Holder A. A. & Schwarz R. T. Structural analysis of the glycosyl-phosphatidylinositol membrane anchor of the merozoite surface proteins-1 and -2 of *Plasmodium falciparum*. Mol. Biochem. Parasitol. 75, 131–143 (1996). 8992312 10.1016/0166-6851(95)02518-9

[b13] Smythe J. A. *et al.* Structural diversity in the *Plasmodium falciparum* merozoite surface antigen 2. Proc. Natl. Acad. Sci. USA 88, 1751–1755 (1991). 2000383 10.1073/pnas.88.5.1751 PMC51102

[b14] Ranford-Cartwright L. C. *et al.* Differential antibody recognition of FC27-like *Plasmodium falciparum* merozoite surface protein MSP2 antigens which lack 12 amino acid repeats. Parasite Immunol. 18, 411–420 (1996). 9229395 10.1046/j.1365-3024.1996.d01-137.x

[b15] Scopel K. K. G., Silva-Nunes M. D., Malafronte R. S., Braga É. M. & Ferreira M. U. Variant-specific antibodies to merozoite surface protein 2 and clinical expression of *Plasmodium falciparum* malaria in rural amazonians. Am. J. Trop. Med. Hyg. 76, 1084–1091 (2007). 17556615

[b16] Eisen D., Billman-Jacobe H., Marshall V. F., Fryauff D. & Coppel R. L. Temporal variation of the merozoite surface protein-2 gene of *Plasmodium falciparum*. Infect. Immun. 66, 239–246 (1998). 9423864 10.1128/iai.66.1.239-246.1998 PMC107883

[b17] Zhang X. *et al.* Solution conformation, backbone dynamics and lipid interactions of the intrinsically unstructured malaria surface protein MSP2. J. Mol. Biol. 379, 105–121 (2008). 18440022 10.1016/j.jmb.2008.03.039 PMC4432223

[b18] MacRaild C. A. *et al.* Conformational dynamics and antigenicity in the disordered malaria antigen merozoite surface protein 2. PLoS ONE 10, e0119899 (2015). 25742002 10.1371/journal.pone.0119899 PMC4351039

[b19] Adda C. G. *et al.* Antigenic characterization of an intrinsically unstructured protein, *Plasmodium falciparum* merozoite surface protein 2. Infect. Immun. 80, 4177–4185 (2012). 22966050 10.1128/IAI.00665-12 PMC3497424

[b20] Taylor R. R., Allen S. J., Greenwood B. M. & Riley E. M. IgG3 antibodies to *Plasmodium falciparum* merozoite surface protein 2 (MSP2): increasing prevalence with age and association with clinical immunity to malaria. Am. J. Trop. Med. Hyg. 58, 406–413 (1998). 9574783 10.4269/ajtmh.1998.58.406

[b21] Ekala M. T., Jouin H., Lekoulou F., Mercereau-Puijalon O. & Ntoumi F. Allelic family-specific humoral responses to merozoite surface protein 2 (MSP2) in Gabonese residents with *Plasmodium falciparum* infections. Clin. Exp. Immunol. 129, 326–331 (2002). 12165090 10.1046/j.1365-2249.2002.01904.x PMC1906438

[b22] Boyle M. J. *et al.* Human antibodies fix complement to inhibit *Plasmodium falciparum* invasion of erythrocytes and are associated with protection against malaria. Immunity 42, 580–590 (2015). 25786180 10.1016/j.immuni.2015.02.012 PMC4372259

[b23] Osier F. *et al.* Opsonic phagocytosis of *Plasmodium falciparum* merozoites: mechanism in human immunity and a correlate of protection against malaria. BMC Med. 12, 108 (2014). 24980799 10.1186/1741-7015-12-108 PMC4098671

[b24] McCarthy J. S. *et al.* A phase 1 trial of MSP2-C1, a blood-stage malaria vaccine containing 2 isoforms of MSP2 formulated with Montanide^®^ ISA 720. PLoS ONE 6, e24413 (2011). 21949716 10.1371/journal.pone.0024413 PMC3176224

[b25] Besteiro S., Michelin A., Poncet J., Dubremetz J.-F. & Lebrun M. Export of a *Toxoplasma gondii* rhoptry neck protein complex at the host cell membrane to form the moving junction during invasion. PLoS Pathog. 5, e1000309 (2009). 19247437 10.1371/journal.ppat.1000309 PMC2642630

[b26] Genton B. *et al.* A recombinant blood-stage malaria vaccine reduces *Plasmodium falciparum* density and exerts selective pressure on parasite populations in a phase 1-2b trial in Papua New Guinea. J. Infect. Dis. 185, 820–827 (2002). 11920300 10.1086/339342

[b27] Genton B. *et al.* Safety and immunogenicity of a three-component blood-stage malaria vaccine (MSP1, MSP2, RESA) against *Plasmodium falciparum* in Papua New Guinean children. Vaccine 22, 30–41 (2003). 14604568 10.1016/s0264-410x(03)00536-x

[b28] Morales R. A. V. *et al.* Structural basis for epitope masking and strain specificity of a conserved epitope in an intrinsically disordered malaria vaccine candidate. Sci. Rep. 5, 10103 (2015). 25965408 10.1038/srep10103 PMC4428071

[b29] Yang X. *et al.* Identification of key residues involved in fibril formation by the conserved N-terminal region of *Plasmodium falciparum* merozoite surface protein 2 (MSP2). Biochimie 92, 1287–1295 (2010). 20542076 10.1016/j.biochi.2010.06.001 PMC2948610

[b30] Zhang X. *et al.* Role of the helical structure of the N-terminal region of *Plasmodium falciparum* merozoite surface protein 2 in fibril formation and membrane interaction. Biochemistry 51, 1380–1387 (2012). 22304430 10.1021/bi201880s

[b31] Flück C. *et al.* Strain-specific humoral response to a polymorphic malaria vaccine. Infect. Immun. 72, 6300–6305 (2004). 15501757 10.1128/IAI.72.11.6300-6305.2004 PMC523016

[b32] Lawrence N., Stowers A., Mann V., Taylor D. & Saul A. Recombinant chimeric proteins generated from conserved regions of *Plasmodium falciparum* merozoite surface protein 2 generate antiparasite humoral responses in mice. Parasite Immunol. 22, 211–221 (2000). 10792760 10.1046/j.1365-3024.2000.00293.x

[b33] Fowkes F., Simpson J. & Beeson J. Implications of the licensure of a partially efficacious malaria vaccine on evaluating second-generation vaccines. BMC Med. 11, 232 (2013). 24228861 10.1186/1741-7015-11-232 PMC4225678

[b34] Tompa P. Intrinsically unstructured proteins. Trends Biochem. Sci. 27, 527–533 (2002). 12368089 10.1016/s0968-0004(02)02169-2

[b35] Adda C. G. *et al.* *Plasmodium falciparum* merozoite surface protein 2 is unstructured and forms amyloid-like fibrils. Mol. Biochem. Parasitol. 166, 159–171 (2009). 19450733 10.1016/j.molbiopara.2009.03.012 PMC2713819

[b36] Chandrashekaran I. R., Adda C. G., MacRaild C. A., Anders R. F. & Norton R. S. Inhibition by flavonoids of amyloid-like fibril formation by *Plasmodium falciparum* merozoite surface protein 2. Biochemistry 49, 5899–5908 (2010). 20545323 10.1021/bi902197x

[b37] Genton B. *et al.* Safety and immunogenicity of a three-component blood-stage malaria vaccine in adults living in an endemic area of Papua New Guinea. Vaccine 18, 2504–2511 (2000). 10775784 10.1016/s0264-410x(00)00036-0

[b38] Balam S. *et al.* *Plasmodium falciparum* merozoite surface protein 2: epitope mapping and fine specificity of human antibody response against non-polymorphic domains. Malar. J. 13, 510 (2014). 25526742 10.1186/1475-2875-13-510 PMC4320585

[b39] Boyle M. J. *et al.* Sequential processing of merozoite surface proteins during and after erythrocyte invasion by *Plasmodium falciparum*. Infect. Immun. 82, 924–936 (2014). 24218484 10.1128/IAI.00866-13 PMC3958018

[b40] Metzger W. G. *et al.* Serum IgG3 to the *Plasmodium falciparum* merozoite surface protein 2 is strongly associated with a reduced prospective risk of malaria. Parasite Immunol. 25, 307–312 (2003). 14507328 10.1046/j.1365-3024.2003.00636.x

[b41] Duah N. O., Miles D. J. C., Whittle H. C. & Conway D. J. Acquisition of antibody isotypes against *Plasmodium falciparum* blood stage antigens in a birth cohort. Parasite Immunol. 32, 125–134 (2010). 20070826 10.1111/j.1365-3024.2009.01165.x PMC2814092

[b42] Tongren J. E., Corran P. H., Jarra W., Langhorne J. & Riley E. M. Epitope-specific regulation of immunoglobulin class switching in mice immunized with malarial merozoite surface proteins. Infect. Immun. 73, 8119–8129 (2005). 16299306 10.1128/IAI.73.12.8119-8129.2005 PMC1307071

[b43] Polley S. D. *et al.* High levels of serum antibodies to merozoite surface protein 2 of *Plasmodium falciparum* are associated with reduced risk of clinical malaria in coastal Kenya. Vaccine 24, 4233–4246 (2006). 16111789 10.1016/j.vaccine.2005.06.030

[b44] Weisman S. *et al.* Antibody responses to infections with strains of *Plasmodium falciparum* expressing diverse forms of merozoite surface protein 2. Infect. Immun. 69, 959–967 (2001). 11159991 10.1128/IAI.69.2.959-967.2001 PMC97975

[b45] Faber B. W. *et al.* Diversity covering AMA1-MSP119 fusion proteins as malaria vaccines. Infect. Immun. 81, 1479–1490 (2013). 23429538 10.1128/IAI.01267-12 PMC3648017

[b46] Etemad B. *et al.* An envelope domain III–based chimeric antigen produced in *Pichia pastoris* elicits neutralizing antibodies against all four dengue virus serotypes. Am. J. Trop. Med. Hyg. 79, 353–363 (2008). 18784226

[b47] Faber B. W. *et al.* Malaria vaccine-related benefits of a single protein comprising *Plasmodium falciparum* apical membrane antigen 1 domains I and II fused to a modified form of the 19-kilodalton C-terminal fragment of merozoite surface protein 1. Infect. Immun. 75, 5947–5955 (2007). 17938224 10.1128/IAI.01804-06 PMC2168333

[b48] Dutta S. *et al.* Overcoming antigenic diversity by enhancing the immunogenicity of conserved epitopes on the malaria vaccine candidate apical membrane antigen-1. PLoS Pathog. 9, e1003840 (2013). 24385910 10.1371/journal.ppat.1003840 PMC3873463

[b49] Saul A. *et al.* Human phase I vaccine trials of 3 recombinant asexual stage malaria antigens with Montanide ISA720 adjuvant. Vaccine 17, 3145–3159 (1999). 10462251 10.1016/s0264-410x(99)00175-9

[b50] Rzepczyk C. M. *et al.* Amino acid sequences recognized by T cells: studies on a merozoite surface antigen from the FCQ-27/PNG isolate of *Plasmodium falciparum*. Immunol. Lett. 25, 155–163 (1990). 1704345 10.1016/0165-2478(90)90108-3

[b51] Rzepczyk C. M. *et al.* Comparative study of the T cell response to two allelic forms of a malarial vaccine candidate protein. J. Immunol. 148, 1197–1204 (1992). 1371134

[b52] Kusi K. A., Faber B. W., Thomas A. W. & Remarque E. J. Humoral immune response to mixed *Pf*AMA1 alleles; multivalent *Pf*AMA1 vaccines induce broad specificity. PLoS ONE 4, e8110 (2009). 19956619 10.1371/journal.pone.0008110 PMC2779588

[b53] Chaudhury S., Reifman J. & Wallqvist A. Simulation of B cell affinity maturation explains enhanced antibody cross-reactivity induced by the polyvalent malaria vaccine AMA1. J. Immunol. 193, 2073–2086 (2014). 25080483 10.4049/jimmunol.1401054 PMC4135178

[b54] Denis J. *et al.* Immunogenicity of papaya mosaic virus-like particles fused to a hepatitis C virus epitope: Evidence for the critical function of multimerization. Virology 363, 59–68 (2007). 17320136 10.1016/j.virol.2007.01.011

[b55] Qian F. *et al.* Immunogenicity of self-associated aggregates and chemically cross-linked conjugates of the 42 kDa *Plasmodium falciparum* merozoite surface protein-1. PLoS ONE 7, e36996 (2012). 22675476 10.1371/journal.pone.0036996 PMC3366955

[b56] Rudra J. S., Tripathi P. K., Hildeman D. A., Jung J. P. & Collier J. H. Immune responses to coiled coil supramolecular biomaterials. Biomaterials 31, 8475–8483 (2010). 20708258 10.1016/j.biomaterials.2010.07.068 PMC3028966

[b57] Kessler M., Goldsmith D. & Schellekens H. Immunogenicity of biopharmaceuticals. Nephrol. Dial. Transplant. 21, v9–v12 (2006). 16959792 10.1093/ndt/gfl476

[b58] Bachmann M. F. & Zinkernagel R. M. Neutralizing antiviral B cell responses. Annu. Rev. Immunol. 15, 235–270 (1997). 9143688 10.1146/annurev.immunol.15.1.235

[b59] Kalthoff C. A novel strategy for the purification of recombinantly expressed unstructured protein domains. J. Chromatogr. B Analyt. Technol. Biomed. Life. Sci. 786, 247–254 (2003). 10.1016/s1570-0232(02)00908-x 12651021

[b60] Brito L. A. & Singh M. Acceptable levels of endotoxin in vaccine formulations during preclinical research. J. Pharm. Sci. 100, 34–37 (2011). 20575063 10.1002/jps.22267

