# Supplementary material for: Strain-transcending immune response generated by chimeras of the malaria vaccine candidate merozoite surface protein 2
Source: Sci Rep. 2016 Feb 11;6:20613. doi: 10.1038/srep20613 (PMC4749986; doi:10.1038/srep20613)
Supplement: Supplementary Information [file srep20613-s1.pdf]

Supplementary Information

Strain-transcending immune response generated by chimeras of the malaria vaccine candidate merozoite surface protein 2

Bankala Krishnarjuna[1+], Dean Andrew[2+], Christopher A. MacRaild[1], Rodrigo A. V. Morales[1], James G. Beeson[2,3,4], Robin F. Anders[5], Jack S. Richards[2,3,4*] and Raymond S. Norton[1*]

[1]Medicinal Chemistry, Monash Institute of Pharmaceutical Sciences, Monash University, Parkville 3052, Australia

[2]Centre for Biomedical Research, Burnet Institute, Melbourne, Victoria 3004, Australia

[3]Department of Microbiology, Monash University, Clayton 3800, Australia

[4]Department of Medicine, University of Melbourne, Parkville, 3052, Australia

[5]Department of Biochemistry and Genetics, La Trobe Institute for Molecular Science, La Trobe University, Melbourne, Victoria 3086, Australia

[+]These authors contributed equally.

*Corresponding authors Raymond S. Norton ray.norton@monash.edu; Jack S. Richards richards@burnet.edu.au

Table S1. Amino acid sequences for the truncated FC27 MSP2 and different MSP2 chimeras. The sequences are colour-coded to match those in Fig. 1B and the repeat sequences are underlined. The N-terminal conserved region is fully conserved across all strains of malaria, while the C-terminal conserved region is conserved except for a single point mutation (Asn to Ser, bold and underlined in the sequence of $V_{FC27}C$), as documented by Fenton et al. and Smythe et al. (9, 10).

MSP2 constructs	Amino acid sequences
NV_{FC27}	GIKNESKYSNTFINNAYNMSIRRSMANEGSNTNSVGANAPNADT<br>IASGSQRSTNSASTSTTNNGESQTTTPTAADTIASGSQRSTNSAST<br>STTNNGESQTTTPTAADTPTATESNSPSPPITTTESSSSGNAPNKT<br>DGKGEESEKQNELNESTEEGPRAPQEPQTAENENPA
$V_{FC27}C$	GNEGSNTNSVGANAPNADTIASGSQRSTNSASTSTTNNGESQTT<br>TPTAADTIASGSQRSTNSASTSTTNNGESQTTTPTAADTPTATES<br>NSPSPPITTTESSSSGNAPNKTDGKGEESEKQNELNESTEEGPRAP<br>QEPQTAENENPAAPENKGTGQHGHMHGSRNNHPQNTSDSQKE<br>CTDGNKENCGAATSLL**S**NSS
$NV_{3D7}V_{FC27}C$	GIKNESKYSNTFINNAYNMSIRRSMAESKPSTGAGGSAGGSAGG<br>SAGGSAGGSAGGSAGSGDGNGADAEGSSSTPATTTTTKTTTTTT<br>TTNDAEASTSTSSENPNHKNAETNPKGKGEVQEPNQANKETQN<br>NSNVQQDSQTKSNVPPTQDADTKSPTAQPEQAENSAPTAEQTES<br>PELQSNEGSNTNSVGANAPNADTIASGSQRSTNSASTSTTNNGES<br>QTTTPTAADTIASGSQRSTNSASTSTTNNGESQTTTPTAADTPTA<br>TESNSPSPPITTTESNSPSPPITTTESSSSGNAPNKTDGKGEESEKQ<br>NELNESTEEGPRAPQEPQTAENENPAAPENKGTGQHGHMHGSR<br>NNHPQNTSDSQKECTDGNKENCGAATSLLSNSS
$V_{3D7}V_{FC27}C$	GESKPSTGAGGSAGGSAGGSAGGSAGGSAGGSAGSGDGNGAD<br>AEGSSSTPATTTTTKTTTTTTTTNDAEASTSTSSENPNHKNAETN<br>PKGKGEVQEPNQANKETQNNSNVQQDSQTKSNVPPTQDADTKS<br>PTAQPEQAENSAPTAEQTESPELQSNEGSNTNSVGANAPNADTI<br>ASGSQRSTNSASTSTTNNGESQTTTPTAADTIASGSQRSTNSAST<br>STTNNGESQTTTPTAADTPTATESNSPSPPITTTESSSSGNAPNKT<br>DGKGEESEKQNELNESTEEGPRAPQEPQTAENENPAAPENKGTG<br>QHGHMHGSRNNHPQNTSDSQKECTDGNKENCGAATSLLSNSS
$V_{3D7}V_{FC27}$	GESKPSTGAGGSAGGSAGGSAGGSAGGSAGGSAGSGDGNGAD<br>AEGSSSTPATTTTTKTTTTTTTTNDAEASTSTSSENPNHKNAETN<br>PKGKGEVQEPNQANKETQNNSNVQQDSQTKSNVPPTQDADTKS<br>PTAQPEQAENSAPTAEQTESPELQSNEGSNTNSVGANAPNADTI<br>ASGSQRSTNSASTSTTNNGESQTTTPTAADTIASGSQRSTNSAST<br>STTNNGESQTTTPTAADTPTATESNSPSPPITTTESSSSGNAPNKT<br>DGKGEESEKQNELNESTEEGPRAPQEPQTAENENPA
$NV_{mFC27}V_{m3D7}C$	GIKNESKYSNTFINNAYNMSIRRSMAESKPSTGAESQTTTPTAAD<br>TIASGSQRSTNSASTSTTNNGESQTTTPTAADTPTATESNSPSPPIT<br>TTESSSSGNAPNKTDGKGEESEKQNELNESTEEGPRAPQEPQTAE<br>NENPAGSGDGNGADAEGSSSTPATTTTTKTTTTTTTTNDAEAST<br>STSSENPNHKNAETNPKGKGEVQEPNQANKETQNNSNVQQDSQ<br>TKSNVPPTQDADTKSPTAQPEQAENSAPTAEQTESPELQSAPEN

	KGTGQHGHMHGSRNNHPQNTSDSQKECTDGNKENCGAATSLL SNSS

Table S2. Molecular masses of different MSP2 constructs and estimated endotoxin levels. The molecular masses were determined using LC-MS and endotoxin levels were estimated using an endotoxin assay kit from GenScript.

MSP2 constructs	Expected mass (kDa)	Observed mass (kDa)	Endotoxin levels (EU/µg)
3D7 MSP2	24189.0	24195.0	0.02
FC27 MSP2	23842.0	23849.0	0.02
NV_{FC27}	17644.2	17654.7	0.08
$V_{FC27}C$	19926.3	19933.6	0.04
$NV_{3D7}V_{FC27}C$	38999.0	39048.2	0.01
$V_{3D7}V_{FC27}C$	34851.4	34908.0	0.02
$V_{3D7}V_{FC27}$	29334.0	29342.0	0.02
$NV_{mFC27}V_{m3D7}C$	32515.6	32530.2	0.03

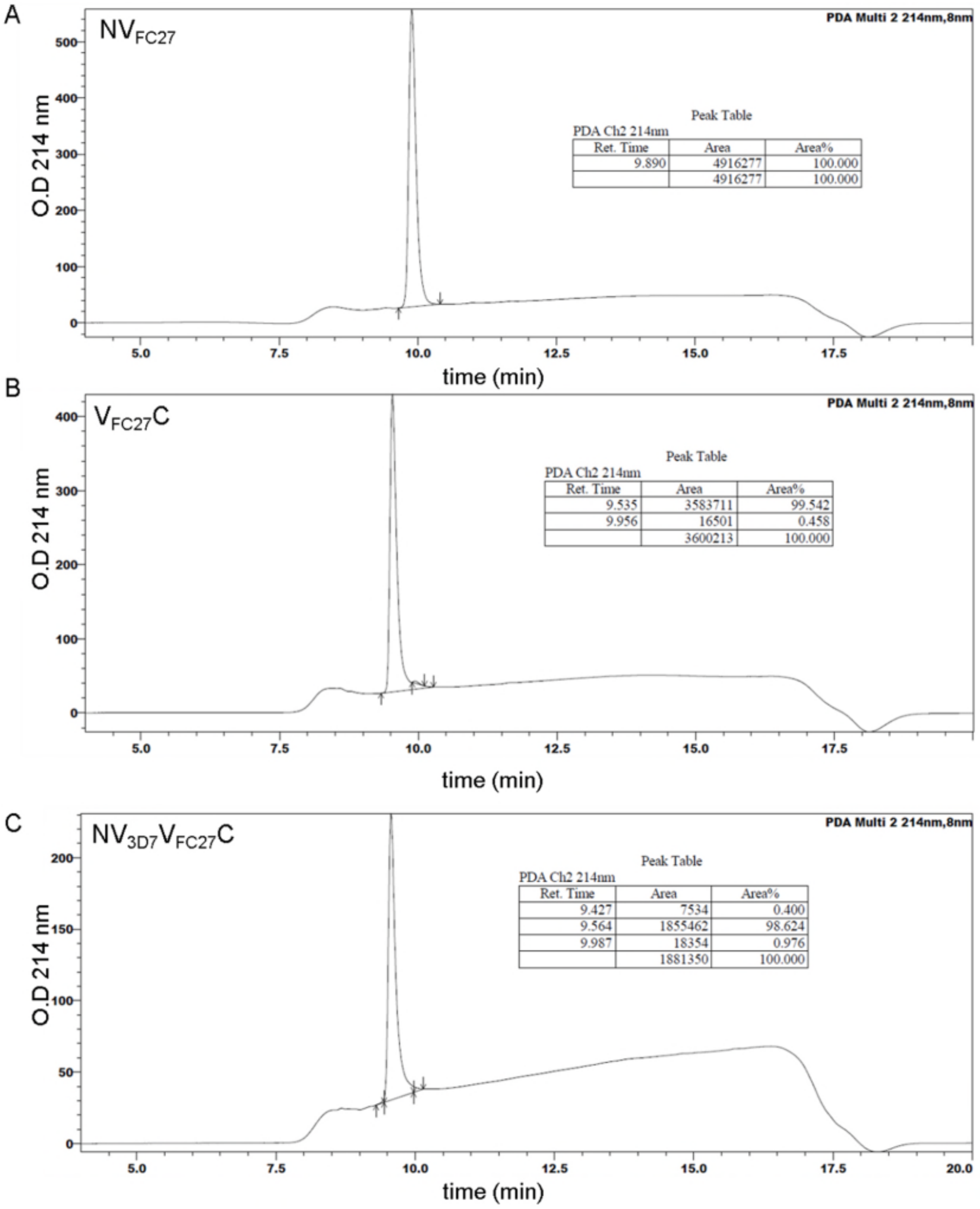

Figure S1 (continued)

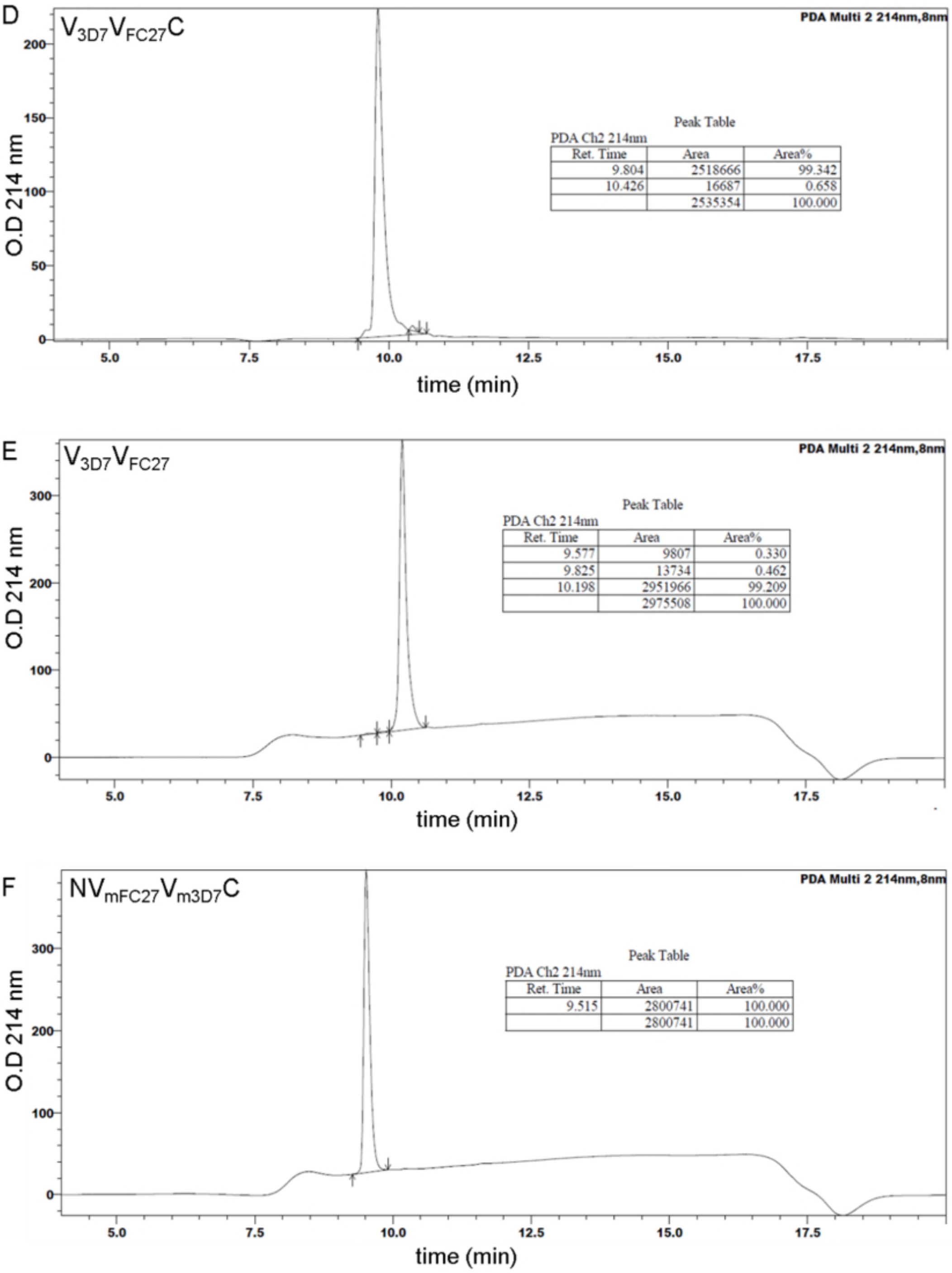

Figure S1. Purity of the MSP2 variants tested by analytical HPLC. The table in each panel shows the purity level of the corresponding MSP2 construct. Samples were assayed on a C4 analytical HPLC column using a gradient of 0-80% acetonitrile containing 0.1% formic acid.

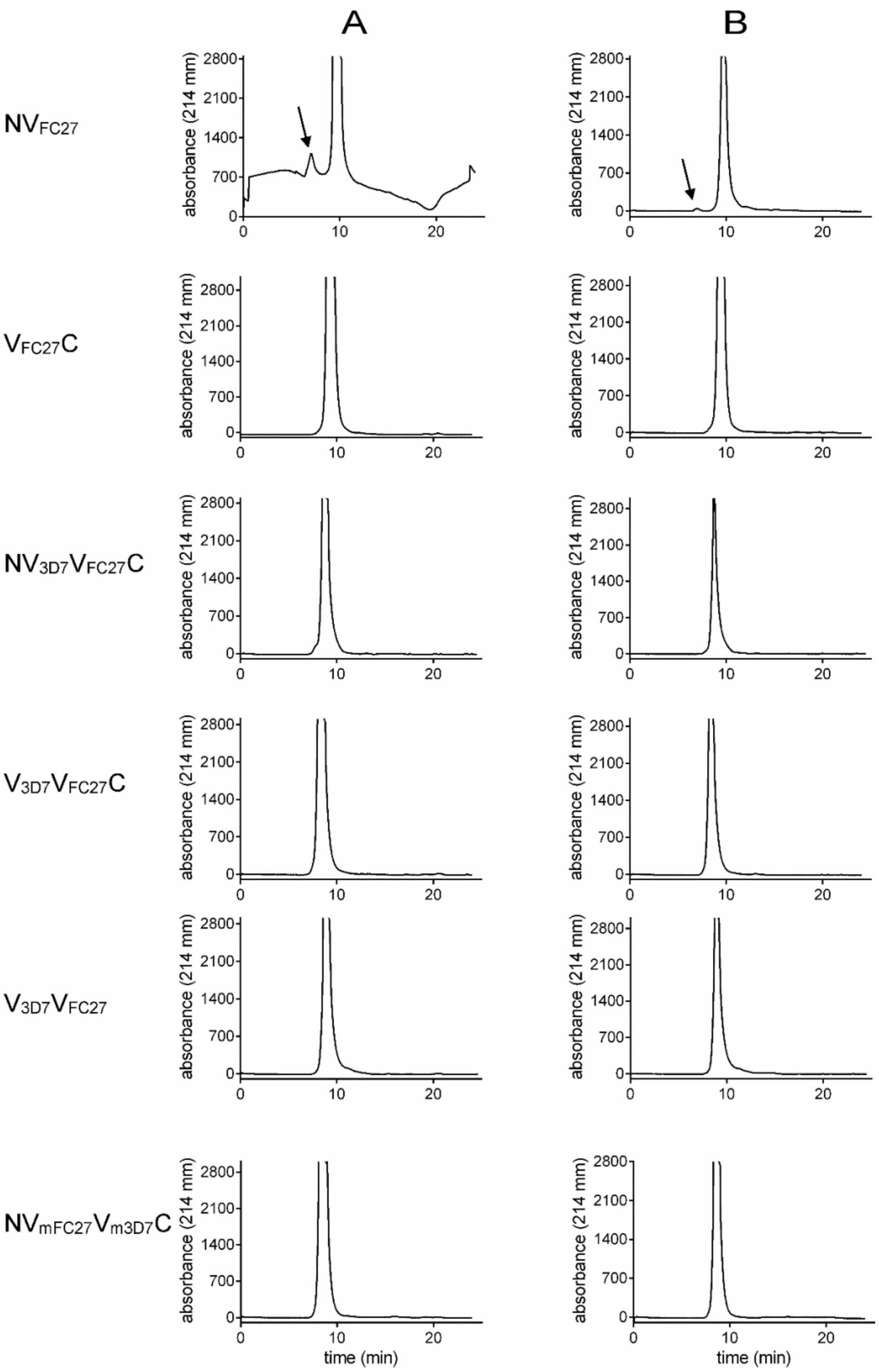

Figure S2. Aggregation propensity of MSP2 constructs (Fig. 1B) as analysed by size-exclusion chromatography. The left panel (A) represents chromatograms of the samples loaded directly onto the size-exclusion column, and the right panel (B) the samples that were preheated before loading onto the size-exclusion column. The arrows indicate aggregated protein in the NV$_{FC27}$ construct.

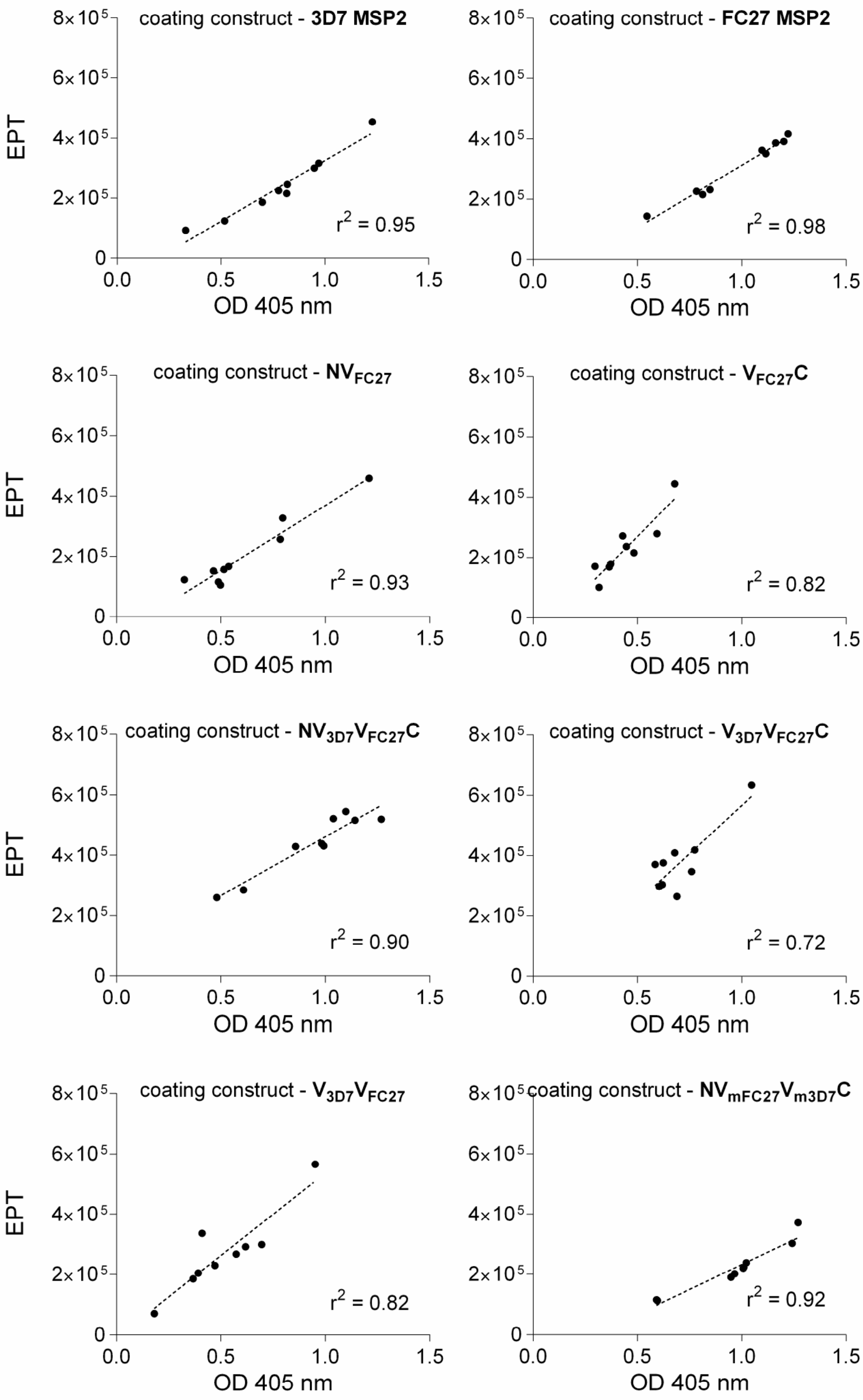

Figure S3. Correlations between the endpoint titres (EPT) and single sera dilations for total IgG responses. The EPT and optical density (OD) were determined using sera from individual mice in each of the immunisation groups against each of the coating antigens (as indicated). The median EPT and median OD for each of these immunisation groups were plotted and the coefficient of determination calculated.

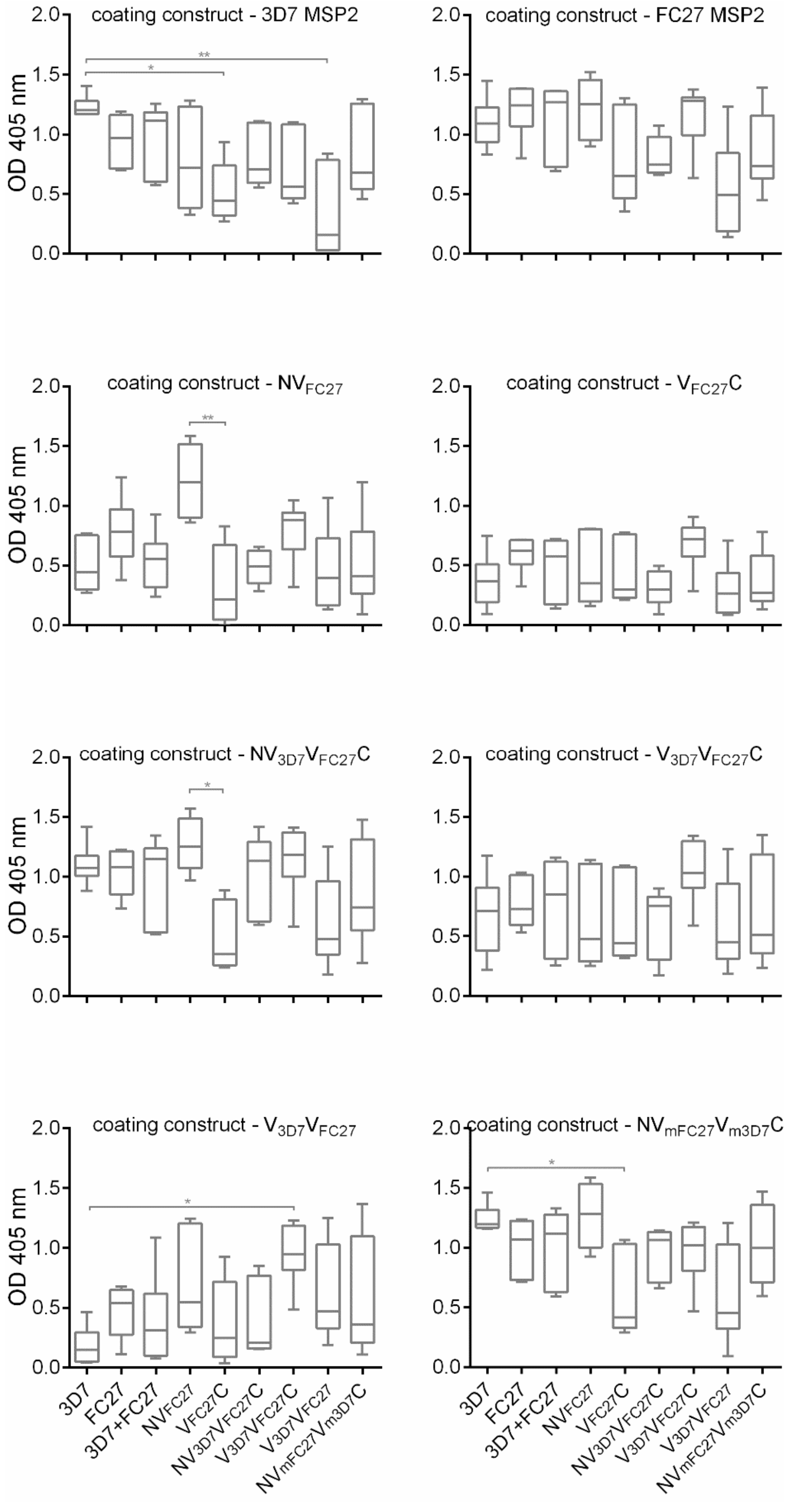

Figure S4. Cross-reactivity of the total IgG raised against different MSP2 constructs (coating construct) with recombinant 3D7 MSP2, FC27 MSP2 and other MSP2 constructs (Fig. 1B). The differences between the groups that are statistically significant (p value ≤0.05) are represented by asterisks (*). OD, optical density.

A

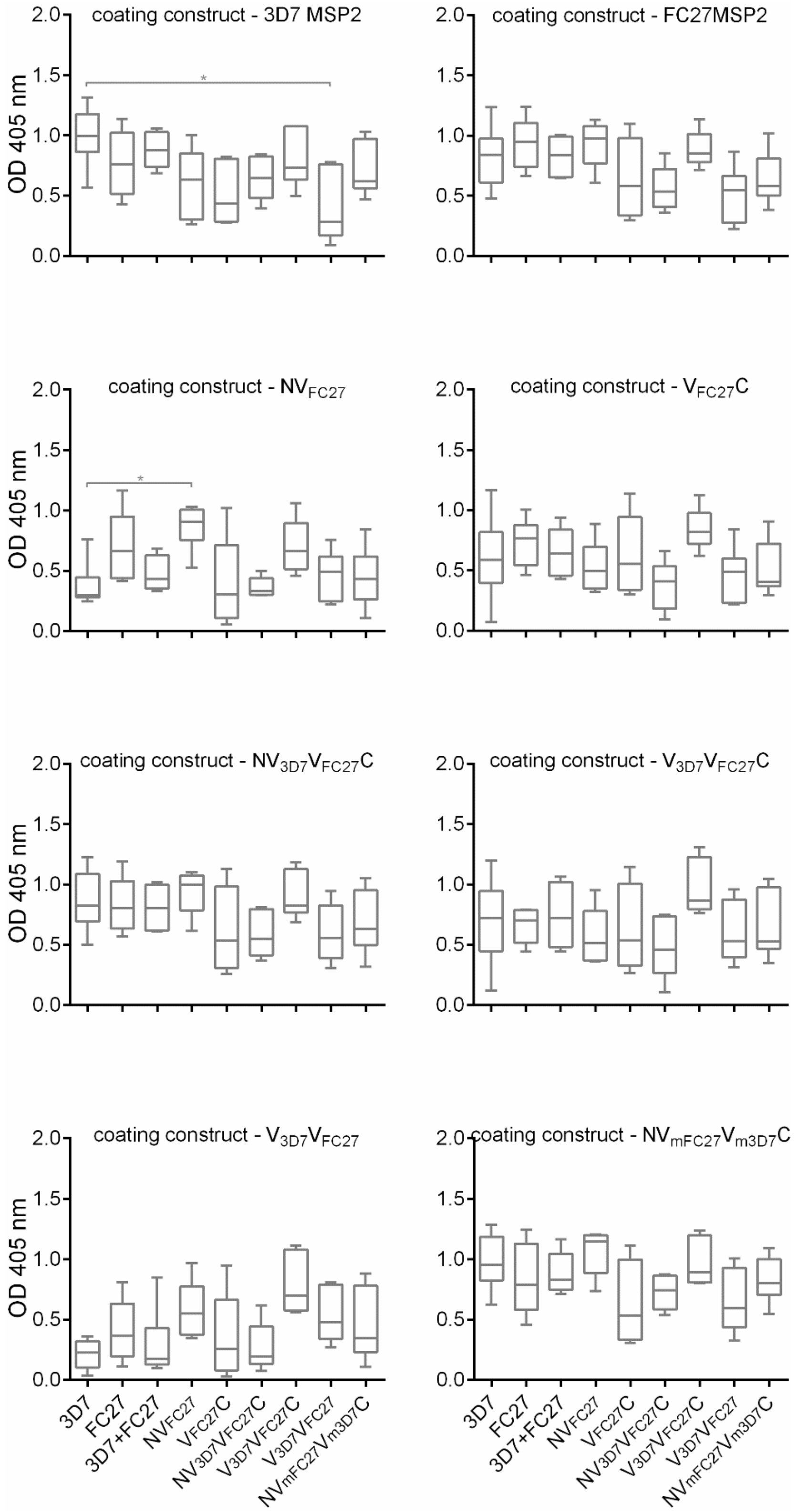

Figure S5 (continued)

B

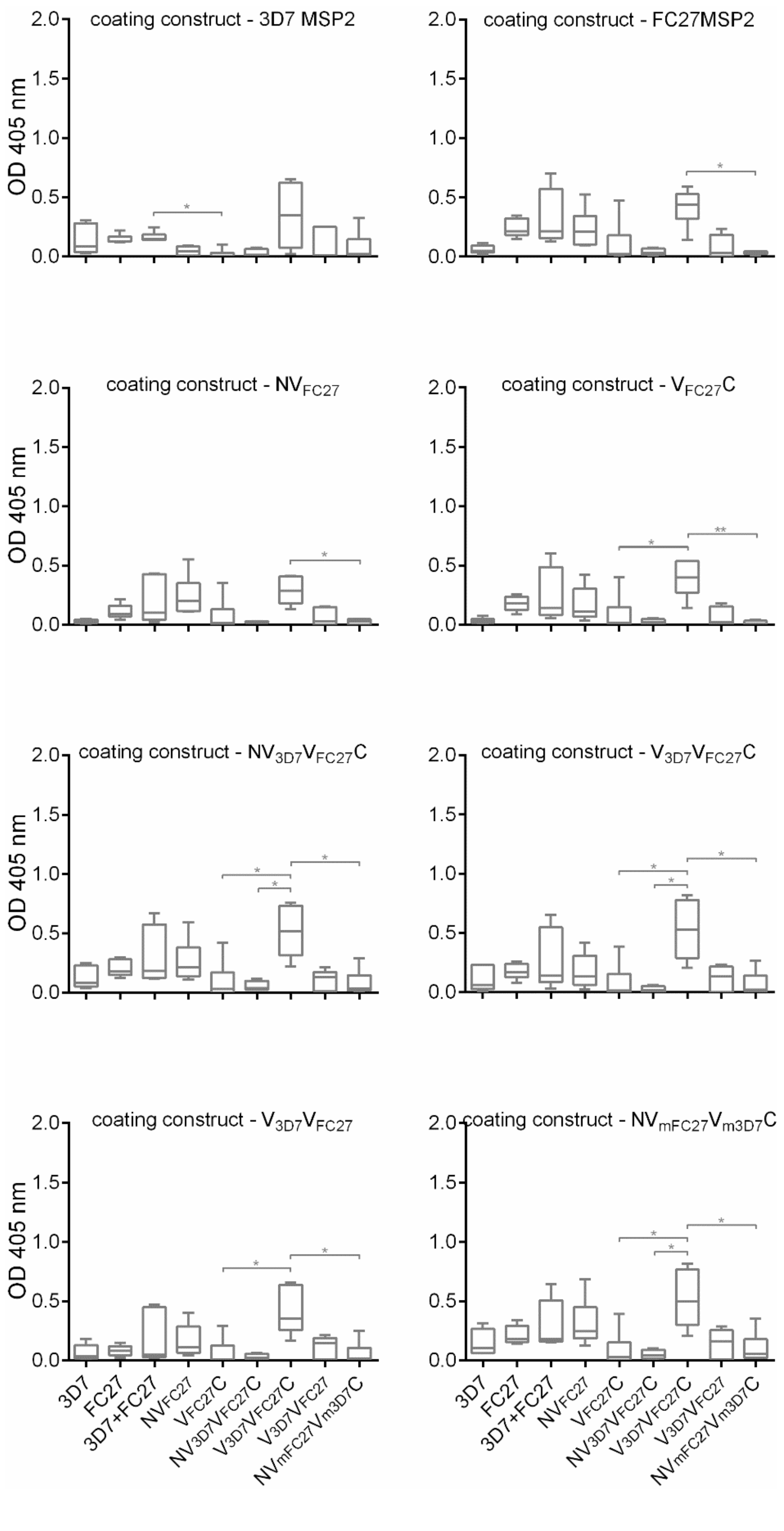

Figure S5 (continued)

C

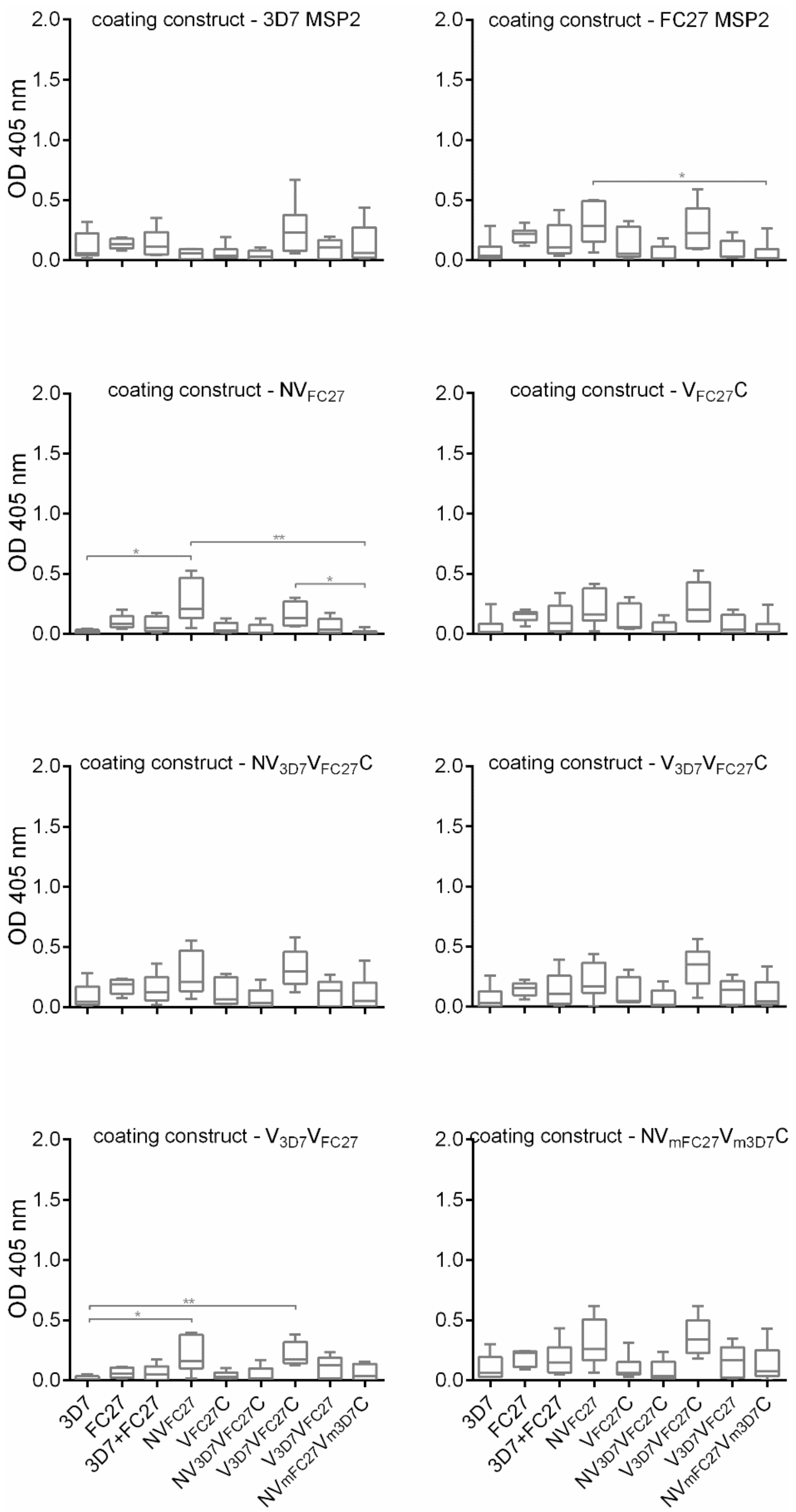

Figure S5 (continued)

D

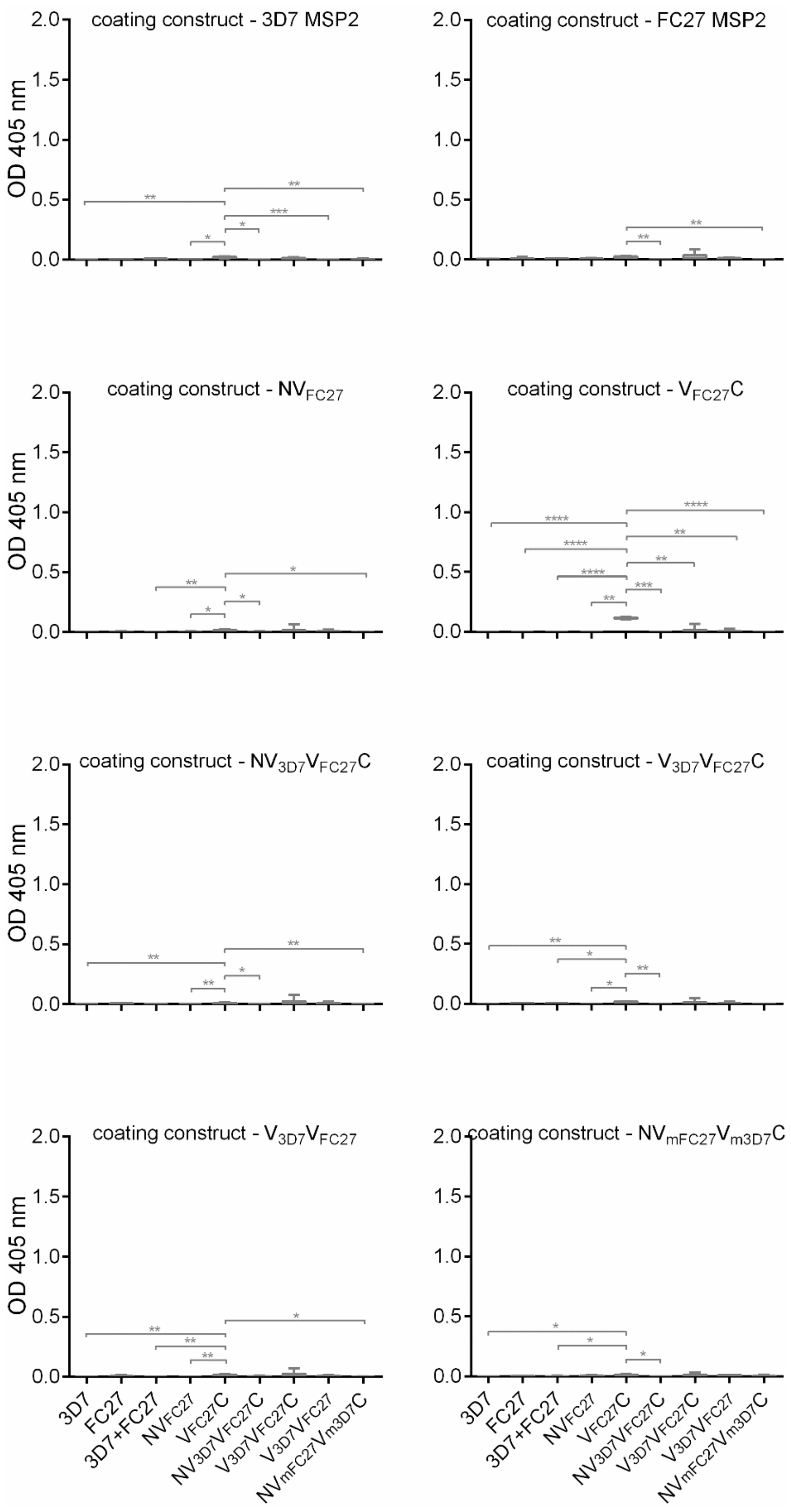

Figure S5. Cross-reactivity of the (A) IgG1, (B) IgG2b, (C) IgGc and (D) IgG3 raised against different MSP2 antigens (coating construct) with recombinant 3D7 MSP2, FC27 MSP2 and other MSP2 constructs. The differences between the groups that are statistically significant are represented as * p≤0.05, ** p≤ 0.01, *** p≤ 0.001 and **** p≤ 0.0001. OD, optical density.
